# Reprogramming dendritic cells to overcome tumor-mediated immune suppression

**DOI:** 10.3389/fimmu.2026.1844446

**Published:** 2026-06-17

**Authors:** Jonaid Ahmad Malik

**Affiliations:** Department of Biomedical Engineering, Indian Institute of Technology, Ropar, India

**Keywords:** dendritic cells, dysfunction, immunotherapy, novel mechanisms, reprogramming, tumor microenvironment

## Abstract

Dendritic cells (DCs) are indispensable orchestrators of antitumor immunity, yet in solid tumors, they frequently undergo profound dysfunction, enabling immune evasion and cancer progression. Recent advances have uncovered unexpected roles of DC subsets in tumor antigen processing, cross-presentation, and immune regulation that were previously underappreciated. In this review, the paper discusses novel mechanisms driving DC impairment, including epigenetic reprogramming, metabolic remodeling, and tumor-derived suppressive networks that converge to skew DC function toward tolerance. It highlights cutting-edge strategies that actively reprogram dysfunctional DCs, such as transcription factor modulation, nanoparticle-based antigen delivery, neoantigen and tumor lysate pulsing, and *in vivo* induction of DC-like phenotypes from malignant or stromal cells. The paper provides a conceptual framework for restoring DC immunogenicity in the tumor microenvironment by integrating these mechanistic insights with translational innovations. This perspective consolidates emerging knowledge and identifies promising therapeutic avenues to enhance the efficacy of DC-based cancer immunotherapy.

## 1 Introduction

Dendritic cells (DCs) in mice and humans are professional antigen-presenting cells (APCs) that activate naïve T cells (1, 2). The specific T cell responses they drive depend on the DC subset and the type of stimulus they encounter. DC subsets develop from committed DC precursors in the bone marrow under the influence of transcription factors and the growth factor Flt3-ligand. These precursors give rise to conventional DCs, which are highly efficient at antigen presentation, and plasmacytoid DCs, formerly known as natural interferon-producing cells, which are the primary source of type I interferons (3). The role of cDC2s in cancer immunology is less well-defined than that of other DC subsets, likely due to their heterogeneity and the limited availability of specific markers for their identification. Nonetheless, cDC2s are important inducers of CD4^+^ T helper cell responses, facilitating effective presentation of tumor antigens on MHC II molecules (4, 5). Secondary lymphoid organs such as the spleen and lymph nodes contain two functionally distinct DC populations: lymphoid-resident DCs, which differentiate locally within these organs, and migratory DCs, which originate in non-lymphoid peripheral tissues and travel to lymph nodes (6). Importantly, the spleen harbors only lymphoid-resident DCs, as it lacks the afferent lymphatics required for migratory DC entry (7).

Anti-tumor CD8^+^ T-cell responses are activated through cross-priming, which requires the acquisition of tumor-derived antigens (Ag) by APCs through multiple mechanisms, including phagocytosis of dead or dying tumor cells, macropinocytosis, or uptake of shed vesicles and soluble antigens released from the tumor microenvironment (TME) (8). The APCs then route the internalized material to a specialized endosomal/lysosomal pathway, enabling the processed peptides to be presented on MHC class I (MHC-I) molecules, a process known as cross-presentation (8–10). DCs are described as the primary cell type capable of cross-presenting cell-associated Ag (10).

Recent clinical studies with naturally circulating DCs, including plasmacytoid DCs (pDCs), represent an exciting development. pDC-based vaccines are particularly interesting, as pDCs are generally thought to suppress T cell responses in tumors. Likewise, DC-derived exosomes (DCexos) have been proposed as cell-free cancer vaccines that may better overcome tumor-induced immunosuppression. However, clinical trials with DCexos have not achieved the anticipated results (11, 12). Recent evidence has revealed the pDC’s role in activating and expanding ICOS-expressing FOXP3+ regulatory T cells within the tumor, shedding light on the mechanisms underlying cancer immune escape (13). These limitations have prompted exploration of alternative strategies to harness DC functionality more effectively. Studies have demonstrated that using a pDC-targeted vaccine to deliver Ag specifically to pDCs induced strong cross-priming and durable CD8 T cell immunity. Surprisingly, cross-presenting pDCs required cDCs to achieve cross-priming *in vivo* by transferring Ag to cDCs (14). Cross-presenting pDCs could not efficiently prime CD8 T cells independently but enabled antigen-naive cDCs to cross-prime CD8 T cells by transferring Ag to cDCs (14). However, antigen presentation by cDC1s alone may be necessary but not sufficient for maximal antitumor immune responses, as critical crosstalk between DC subsets can significantly alter biological outcomes and likely contributes to tumor-specific immunity in ways that remain incompletely characterized (15).

Various approaches have been used to modify DC functionality to enhance antitumor responses. For example, the *in vivo* requirement for cDC–pDC cross-talk generates antitumor immunity against tumor-derived Ag, with or without the TLR9 agonist CpG. Transferring wild-type pDCs into TLR9-deficient mice demonstrated that TLR9 expression in pDCs is sufficient for CpG to function effectively as an adjuvant. These results indicate that CpGs’ efficacy in cancer immunotherapy relies on cross-talk between pDCs and specific cDC subsets (16).

Stimulation of DCs with various maturation signals leads to increased expression of specific intracellular and surface molecules that facilitate their migration to secondary lymphoid organs and enable them to deliver signals 1, 2, and 3 to T cells (17). The local microenvironment strongly influences the activation and maturation of DCs. Individual factors or combinations can inhibit or reverse it, generating DC subsets with tolerogenic or immunosuppressive functions (17, 18). T-cell priming may occur within the TME or in the draining lymph nodes following the migration of tumor antigen–bearing DCs. However, findings vary across studies regarding which DC subsets transport antigen to lymph nodes. For instance, migratory cDC1 has been shown to deliver antigens to the draining lymph nodes in the B16 melanoma model (19, 20). In contrast, in other systems, both cDC1 and cDC2 have been reported to transport antigen to lymph nodes (20). Thus, T-cell priming within the TME largely depends on the specific DC subsets present and which carry the principal tumor antigens for presentation, particularly to CD8^+^ T cells mediating cytotoxicity against cancer cells. The migratory capacity of these DCs and the extent to which the TME suppresses their activation represent a critical barrier (21).

Therefore, it is essential to delineate these mechanisms to develop strategies that enhance antigen presentation, reprogram immunosuppressive DCs into immunostimulatory states, and ultimately strengthen antitumor immunity. This review provides an integrated “problem–mechanism–strategy–translation” framework for reprogramming solid tumor dendritic cells (DCs). The paper highlights emerging evidence across epigenetic, metabolic, and tumor-derived suppressive networks that converge to impair DC function, and highlights therapeutic strategies designed to restore their immunogenic potential. By connecting mechanistic insight with translational outcomes, we aim to delineate rational avenues to optimize DC-based cancer immunotherapy.

## 2 Dendritic cell dysfunction in solid tumors

DCs recognize and process tumor antigens, presenting them to T cells to initiate an antitumor immune response (17, 22). Dysfunction of DCs impairs this process, which can promote cancer progression and metastasis. In solid tumors, DCs possess significant defects in both phenotype and function, thereby weakening the immune response against tumors (23). This involves the buildup of immature or tolerogenic dendritic cell populations, decreased expression of co-stimulatory molecules, impaired antigen presentation, and reduced secretion of pro-inflammatory cytokines. As a result, priming of tumor-specific T cells is hindered, facilitating immune evasion and tumor progression (22, 24). Dendritic cells serve as an essential link between the innate and adaptive immune systems, and it is vital to understand how this connection is altered in the TME to create effective immunotherapies (25, 26).

Immature DCs, which are highly efficient at engulfing and processing antigens, reside in peripheral tissues. Upon encountering danger-associated molecular patterns (DAMPs) or pathogen-associated molecular patterns (PAMPs), they undergo maturation (27, 28). Upon encountering these danger signals, DCs upregulate co-stimulatory molecules (CD80, CD86, and CD40), chemokine receptors such as CCR7, and secrete pro-inflammatory cytokines, and migrate to the lymph nodes to prime T cells. T cell activation is orchestrated through three essential signals: antigen presentation via MHC molecules (signal 1), co-stimulatory interactions (signal 2), and cytokine-mediated polarization (signal 3) (27, 29, 30). In contrast, antigen presentation in the absence of signal 2 and signal 3 induces T cell tolerance. In the TME, both tumor cells and various immunosuppressive cells contribute to the suppression of DC function and hamper effective antitumor immune responses (27, 31–33). Various interrelated factors contribute to DC dysfunction in the TME, including cytokine-mediated suppression, low oxygen levels, and metabolic changes, each of which will be elaborated on below.

TGF-β, IL-10, and VEGF impair DC maturation and promote Treg expansion, reinforcing an immunosuppressive TME. Research has shown that CD4^+^CD25^+^FoxP3^+^ regulatory T cells in tumor-bearing hosts inhibit DC activity by suppressing NF-κB signaling. This leads to reduced expression of co-stimulatory molecules (CD80, CD86, CD40) and diminished secretion of TNF-α, IL-12, and CCL5/RANTES. The inhibitory effect is mediated through TGF-β and IL-10 and is linked to the activation of Smad and STAT3 signaling pathways (34). High levels of TGFβ1 are observed in immune cells in close contact with Treg cells within the lymphoid stroma, supporting the idea that TGFβ1 acts as an immunosuppressive factor in the tumor stroma (35). Similarly, lung carcinoma cells (LCCs) reduced CD86 expression and TNF-α and IL-12p70 production in mature DCs. In addition, LCCs induced mature DCs to produce TGF-β1. These TGF-β1-producing DCs were inefficient at activating naive CD4^+^ T cells or supporting their proliferation and differentiation into Th1 (IFN-γ^+^) effectors. Instead, they showed an enhanced capacity to generate CD4^+^CD25^+^FoxP3^+^ regulatory T cells, suppressing T cell proliferation (36). Evidence suggests that DC dysfunction in these tumors may be partly driven by elevated TGF-β signaling (37). Targeting TGF-β pathways in the TME may potentially reverse this immunosuppressive reprogramming and restore DC functionality.

In addition to TGF-β, the DC dysfunction in cancer is closely linked to increased levels of cytokines such as interleukin (IL)-6 and IL-10, decreased IL-12 production, and enhanced activation of signal transducer and activator of transcription 3 (STAT3) (38–40). STAT3 mediates extensive crosstalk between tumor cells and the immune microenvironment, thereby driving tumor-induced immunosuppression (40). IL-10, from both external and tumor sources, downregulates CD40 and IL-12 production in DCs, while FLT3L and CD40L treatment restores DC resistance to tumor-induced CD40 suppression (40). Activated B cells with low or absent FcγRII expression suppress T-cell activity through IL-10–dependent pathways, creating conditions that support tumor progression. These findings suggest that therapies targeting the CD95–CD95L axis and IL-10 production may help re-establish effective immune responses, though further validation is needed (41). IL-10 exposure during late-stage culture markedly impairs the ability of immature DCs to induce CD4^+^ T cell responses in a dose-dependent manner (42).

Moreover, hypoxia is another mediator that makes DCs dysfunctional. A hallmark of the TME, hypoxia, drives vascular endothelial growth factor (VEGF) signaling through direct and indirect mechanisms. Hypoxia directly induces VEGF expression via hypoxia-inducible factors (HIFs), while indirectly, VEGF impairs DC maturation and function by engaging VEGF receptors and co-receptors on the cell surface (43, 44). Thyroid tumorigenesis is influenced by VEGF and various DCs subsets. In thyroid cancer, VEGF expression promotes neovascularization while suppressing DC function (45). Similarly, in colorectal cancer, the pDC compartment is both quantitatively and functionally compromised, potentially correlating with disease stage and elevated VEGF levels (46). Cytokines such as IL-10, VEGF, and TGF-β, as discussed above, released within the TME have been shown to contribute to DC tolerance, which may impair the activation of CD4^+^ and CD8^+^ T cells (47). Targeting immunosuppressive cytokine pathways may enhance the host immune response against cancer and improve prognosis in patients with solid tumors.

Rapid proliferation of solid tumors often outstrips their blood supply, creating a hypoxic microenvironment. This oxygen deficiency triggers the Warburg effect and elevates the production of reactive oxygen species (ROS). Both hypoxia and ROS alter immune cell behavior in the TME, impairing immune function (48). Hypoxic conditions within the TME have been shown to reprogram DCs, driving T cell polarization toward a tumor-suppressive Th17 phenotype. Multiple studies provide evidence that hypoxia and ROS act as interdependent regulators of DC function within the TME (48). Hypoxia suppresses the expression of key differentiation and maturation markers (CD1a, CD40, CD80, CD83, CD86, and MHC class II) induced by lipopolysaccharide (LPS), thereby diminishing the ability of DCs to stimulate T-cell functions (49). In tumor samples from patients with HCC, hypoxic regions showed a marked accumulation of Tregs and cDC2 and reduced numbers of CD8^+^ T cells. Under hypoxic conditions, cDC2 also displayed significantly decreased HLA-DR expression, which may contribute to their increased interactions with Tregs (22, 50). Moreover, it is well established that HIF-1α is a central regulator of cellular adaptation to hypoxia (22, 51).

Lipids serve as essential energy reserves and fundamental structural elements of cellular membranes, while also participating in signaling pathways regulating diverse cell types’ functions (52, 53). DCs from tumor-bearing mice and cancer patients exhibit elevated triglyceride levels compared to DCs from tumor-free mice and healthy individuals. Recent findings indicate that this lipid buildup results from enhanced uptake of extracellular lipids driven by the upregulation of scavenger receptor A (53). DCs with elevated lipid content showed impaired ability to stimulate allogeneic T cells and to present tumor-associated antigens. However, their expression of MHC and co-stimulatory molecules was comparable to that of DCs with normal lipid levels (53). Moreover, aberrant tumor cell metabolism reshapes the TME, leading to hyperglycolysis, lactate and lipid accumulation, acidification, and tryptophan depletion. These alterations impair DC function and contribute to tumor immune evasion (54). In murine DCs, TLR stimulation induces a marked shift toward aerobic glycolysis, resembling the Warburg effect observed in cancer cells. This metabolic reprogramming is driven by the phosphatidylinositol 3′-kinase/Akt pathway, counteracted by AMP-activated protein kinase (AMPK), and is essential for DC maturation (55). DC activity is compromised in the TME, largely due to hypoxia and altered metabolic programming. Altered glycolysis contributes to DC dysfunction, impairing their ability to present antigens and activate T cells (56). Since DC maturation and activity are critical for initiating anticancer immunity by stimulating CD8^+^ T cells, strategies that restore DC function are of great therapeutic interest. The combined effects of cytokine-mediated suppression, low oxygen levels, and metabolic changes create an extremely immunosuppressive TME. This environment impairs DCs and prevents the maintenance of a robust anti-tumor immune response.

## 3 Mechanisms to reprogram dysfunctional DCs

**Figure 1:**
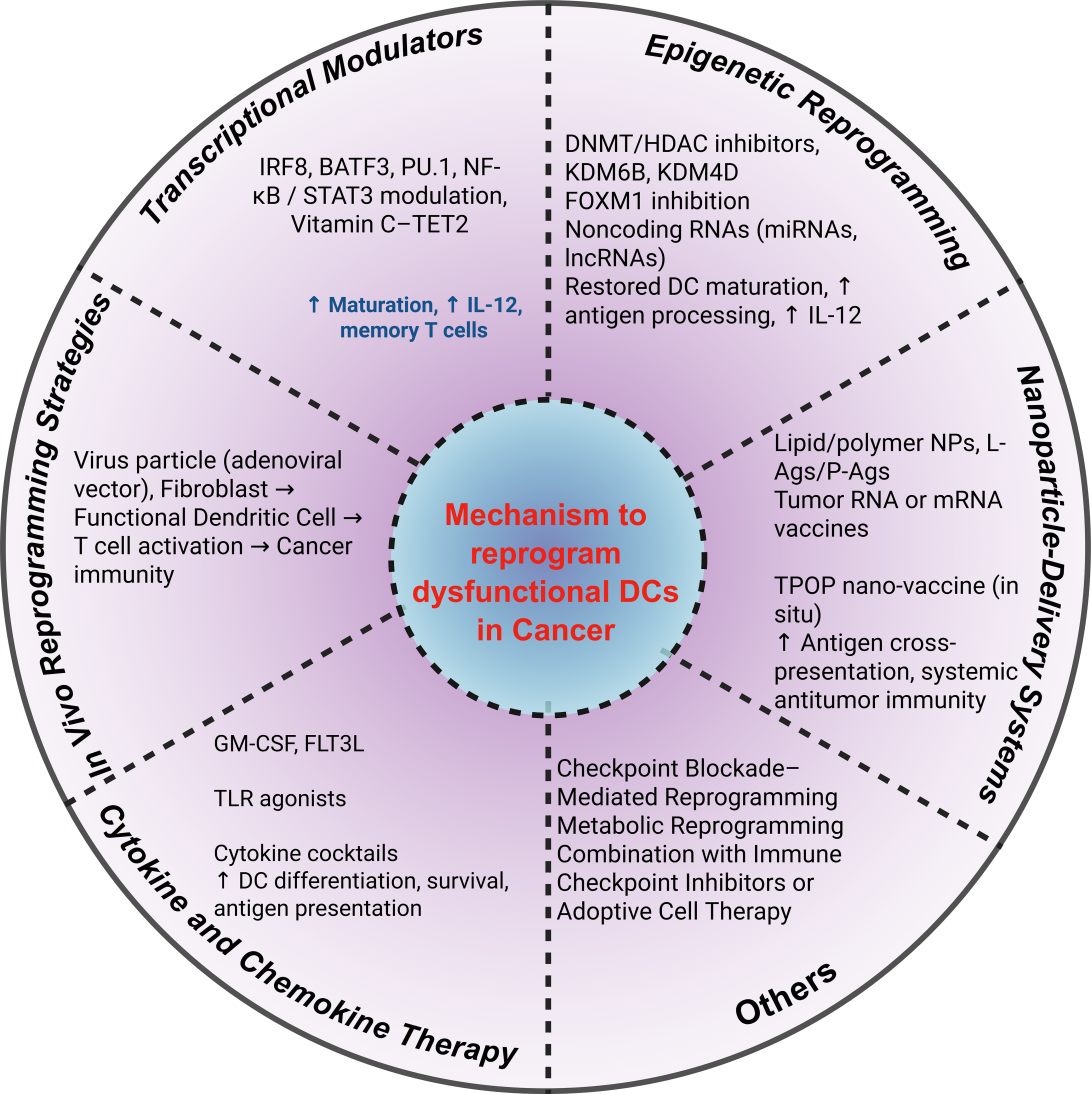
Mechanisms to reprogram dysfunctional DCs in cancer. Multiple strategies have been developed to restore the function of immunosuppressed DCs within the TME. Transcriptional modulators (IRF8, BATF3, PU.1, NF-κB/STAT3 modulation, vitamin C–TET2) enhance DC maturation and IL-12 secretion, improving T cell priming. *In vivo* reprogramming strategies, such as adenoviral delivery of transcription factors or converting fibroblasts and tumor cells into induced DCs, generate functional antigen-presenting cells that activate antitumor immunity. Epigenetic reprogramming (DNMT/HDAC inhibitors, histone demethylases, FOXM1 inhibition, and noncoding RNAs) restores antigen processing and pro-inflammatory cytokine production. Cytokine and chemokine therapy (GM-CSF, FLT3L, TLR agonists, cytokine cocktails) promotes DC differentiation, survival, and antigen presentation. Nanoparticle-based delivery systems (lipid/polymer nanoparticles, tumor RNA or mRNA vaccines, TPOP nanovaccine) enhance antigen cross-presentation and systemic antitumor responses. Additional mechanisms include checkpoint blockade–mediated DC reprogramming, metabolic reprogramming, and integration with immune checkpoint inhibitors or adoptive cell therapies. Collectively, these strategies aim to reverse DC dysfunction, strengthen T cell activation, and improve the efficacy of cancer immunotherapy.

Multiple strategies have been explored to reprogram immunosuppressed DCs within the TME, including modulation of transcription factors (IRF8, BATF3, PU.1, NF-κB), small molecules, CRISPR-Cas9, viral vectors, and epigenetic interventions such as histone modifications and DNA methylation. Additional approaches include cytokine and chemokine signaling (e.g., TLR agonists, GM-CSF, FLT3L), nanoparticle-based delivery of antigens, adjuvants, or mRNA, and *in vivo* reprogramming strategies that convert tumor cells into DC-like cells or expand DCs *in situ*. Collectively, these strategies aim to restore DC functionality, enhance T cell priming, and strengthen antitumor immunity. The following section discusses key mechanisms to reprogram DCs to enhance their function in cancer immunotherapy (**Figure 1**).

### 3.1 Transcriptional modulators

*DCs* are central to immune coordination, functioning as professional APCs and crucial sentinels of tissue integrity. By linking innate and adaptive immunity, DCs act as gatekeepers of antitumor immunity, initiating and modulating responses against tumor antigens (57). Cooperative activity of specific transcription factor triads governs DC lineage diversification, with PU.1–IRF4–PRDM1 driving pro-inflammatory cDC2B-like programs and SPIB–IRF8–IKZF2 promoting immature lymphoid DC fates. These reprogrammed subsets exhibit distinct transcriptomic identities and employ complementary antitumor mechanisms, highlighting opportunities for tailored DC-based immunotherapies (58). Cancer sera simultaneously suppress NF-κB and STAT3 signaling, indicating that inhibiting these pathways may underlie DC dysfunction in the tumor setting (59). Activation of NF-κB represents a central event in multiple pathways governing DC maturation and activation. Importantly, cytokine cocktail–driven DC maturation consistently requires NF-κB signaling (60, 61). Human monocyte-derived DCs matured with cytokine cocktails and transfected with constitutively active IKK mutants showed elevated maturation markers and sustained cytokine secretion, including prolonged IL-12p70 production. Importantly, these caIKK-transfected DCs induced cytotoxic T cells with a memory-like CD27^+^ phenotype, superior recall expansion, and enhanced lytic activity (61). Vitamin C, acting as a cofactor for TET enzymes, promotes active DNA demethylation at NF-κB/p65 binding sites during DC maturation. This process enhances antigen presentation and immune-related gene expression, with the p65–TET2 interaction shown to be essential, as confirmed by pharmacological inhibition (62). Transcription factors such as PU.1, IRF4, PRDM1, and others represent promising candidates for reprogramming DCs within the TME to strengthen antitumor immunity. Future studies should focus on identifying key targets that facilitate the shift of DCs from an immunosuppressive to an immunoreactive state, thereby supporting T cell activation and effective antigen presentation.

### 3.2 *In vivo* reprogramming strategies

Several studies have employed diverse strategies to reprogram DCs within the TME or to engineer cancer cells into APC-like phenotypes, aiming to strengthen antitumor immune responses and improve the efficacy of cancer immunotherapy. Reprogramming strategies that simultaneously limit oncogenic potential while enhancing immune activation in tumor cells hold promise for cancer immunotherapy. Central to this process are cDC1s, which, within the TME, cross-present tumor antigens to CD8^+^ T cells, a key step in initiating effective antitumor immunity (4, 63–65). Ascic et al. introduced an *in vivo* strategy to reprogram malignant cells into DC-like cells through adenoviral delivery of the transcription factors PU.1, IRF8, and BATF3. This reprogramming endowed tumor cells with DC-associated properties, enabling them to process and present antigens and elicit tumor-specific T cell responses (66). Similarly, Zimmermannova et al. explored reprogramming cancer cells into tumor-derived APCs (tumor-APCs). Mouse and primary human cancer cells engineered with the transcription factors PU.1, IRF8, and BATF3 developed a stable DC-like phenotype, gained the capacity to present endogenous tumor antigens, activated CD8^+^ T cell effector functions, and exhibited reduced tumorigenicity. In murine models, intratumoral delivery of tumor-APCs suppressed tumor growth, prolonged survival, and potentiated responses to immune checkpoint blockade (63). In addition, Rosa et al. demonstrated that forced expression of PU.1, IRF8, and BATF3 could reprogram both mouse and human fibroblasts into induced DCs (iDCs). These iDCs closely resembled conventional type 1 DCs, displaying antigen uptake and presentation, while murine iDCs also exhibited the ability to cross-present antigens to CD8^+^ T cells (67). Utilizing the genetic engineering that converts tumor cells into DC-like phenotypes represents a promising avenue for cancer immunotherapy. *In situ* reprogramming could enhance MHC-I–mediated presentation of endogenous tumor antigens, activating CD8^+^ T cells for targeted tumor elimination. Such approaches hold strong potential to reverse tumor cells into functional DC-like states and boost antitumor immunity.

### 3.3 Epigenetic reprogramming

Epigenetic changes, including DNA methylation, histone modifications, and regulation by noncoding RNAs, play a key role in controlling human gene expression and have been shown to effectively contribute to the reprogramming of the tumor immune microenvironment (TIME) (68). In recent years, epigenetic-based therapies have gained attention as promising strategies in cancer treatment, particularly for enhancing the efficacy of cancer vaccines. For instance, upon lipopolysaccharide (LPS) stimulation, DCs activate histone demethylases (HDMs) such as KDM6B (JMJD3) and KDM4D (JMJD2D), which remove the repressive histone marks H3K27me3 and H3K9me3. This process facilitates the transcription of pro-inflammatory genes and promotes inflammatory responses (69, 70). Thus, intratumoral activation of histone-modifying enzymes, such as histone demethylases KDM6B and KDM4D, may be pivotal in enhancing local DC activity. Another illustrative example is the small-molecule inhibitor EPZ004777, which reduces H3K79me2 levels and has been shown to improve DC function within the TME. This effect is partly mediated through downregulating the forkhead box transcription factor M1 (FOXM1). This proliferation-associated regulator drives tumorigenesis by controlling the transcription of target genes across multiple cell types, including DCs (70). Several immune cell populations, including DCs and natural killer (NK) cells, are regulated by histone methylation in the context of cancer (68). The FOXM1 has emerged as a critical regulator of bone marrow-derived dendritic cell (BMDC) maturation and function in pancreatic and colon cancers. Studies demonstrate that FOXM1 impairs BMDC maturation, reduces their ability to drive T-cell proliferation, and lowers interleukin-12 (IL-12) p70 secretion in tumor-bearing mice, thereby contributing to immune suppression within the TME (71).

Epigenetic modulators have emerged as promising agents that enhance tumor immunogenicity through multiple coordinated mechanisms. These include reactivating transcriptionally silenced tumor-associated antigens, enhancing the presentation of neoantigens by upregulating MHC processing pathways, and promoting immunogenic cell death (ICD) (72, 73). The induction of ICD further enriches the repertoire of tumor-derived immunogens, thereby facilitating efficient cross-priming of anti-tumor T cells and sensitizing malignant cells to immune-mediated elimination. These properties position epigenetic modulators as valuable candidates for integration into combinatorial immunotherapy strategies, where they may substantially augment therapeutic efficacy (72). Pharmacological agents that modulate the cellular epigenome to regulate gene expression and cellular function. This category encompasses inhibitors targeting DNA methyltransferases (DNMTs), DNA demethylases, histone deacetylases (HDACs), histone acetyltransferases (HATs), histone methyltransferases (HMTs), histone demethylases (HDMs), and other epigenetic regulators. Beyond enzymatic targets, non-coding RNAs such as microRNAs (miRNAs) and long non-coding RNAs (lncRNAs) also serve as critical epigenetic modulators, orchestrating diverse biological processes. Carcinogenesis and immune regulation fall under their influence, underscoring their therapeutic relevance in cancer treatment (72, 74, 75). Therefore, epigenetic modulators or related agents have the potential to reverse the immunosuppressive state of DCs within the TME. By reprogramming DCs, these agents could restore their antigen-presenting capacity, leading to enhanced expression of MHC class I molecules and more efficient presentation of tumor antigens to T cells. Such reactivation of DC function may significantly improve the efficacy of immunotherapeutic interventions in cancers.

## 4 Translational applications and advancements

DCs can be harnessed for cancer vaccination through several strategies (1): non-targeted peptide, protein, or nucleic acid vaccines that DCs capture *in vivo* (2), vaccines in which antigens are conjugated to anti-DC antibodies for targeted delivery, and (3) *ex vivo*–generated DCs that are preloaded with antigens before administration. Many of these approaches are currently under evaluation in clinical trials (76). DC immunotherapy has demonstrated considerable potential in preclinical cancer models. However, its clinical applicability relies on outcomes from human studies (77). Preclinical studies demonstrate that DCs can be engineered to enhance antigen presentation, enabling robust immune responses even against weakly immunogenic antigens. This property is particularly valuable in the context of tumor vaccination, as it enables overcoming immune tolerance to tumor-associated antigens (76, 77). Various translational approaches currently utilized are outlined below.

### 4.1 Targeting DCs with neoantigens

Neoantigens represent highly attractive targets for DC–based vaccines, as they are uniquely expressed by tumor cells and evade central tolerance. Only a limited number of clinical trials have explored neoantigen-loaded DC vaccines. Importantly, a case of complete tumor regression was reported in a patient with metastatic gastric cancer following treatment with a monocyte-derived dendritic cell (Neo-MoDC) vaccine in combination with immune checkpoint inhibitors (ICIs), highlighting the therapeutic potential of this approach (78). Neoantigen-loaded DC vaccines can generate strong immune responses against gastric cancer–specific peptides, promoting tumor cell lysis and impeding or reversing disease progression. This approach holds substantial promise as a therapeutic strategy for patients with gastric cancer (79).

Recent advances have focused on developing improved strategies for cell-free vaccines, including exosome-based nanoplatforms for cancer immunotherapy and personalized nanotechnology. Such approaches offer a promising pathway to generate individualized nanovaccines suitable for clinical application rapidly (80, 81). Incorporating neoantigens into DCs has been reported as an effective strategy to reprogram their function, improve tumor antigen presentation, and enhance immune activation. A nanovaccine platform has been developed using DC–derived exosomes as carriers, combined with patient-specific neoantigens, to enable individualized immunotherapy. This system demonstrated efficient cargo loading and sustained delivery to lymph nodes, leading to robust, antigen-specific, broad-spectrum T-cell and B-cell immune responses while maintaining high biosafety and biocompatibility (80). Advancing DC-based immunotherapy requires developing new approaches to enhance antigen processing, particularly for tumor-associated antigens. This includes exploring combination regimens and alternative pulsing platforms designed further to augment the immunogenicity of DCs in cancer therapy.

### 4.2 Targeting DCs with tumor lysates

**Figure 2:**
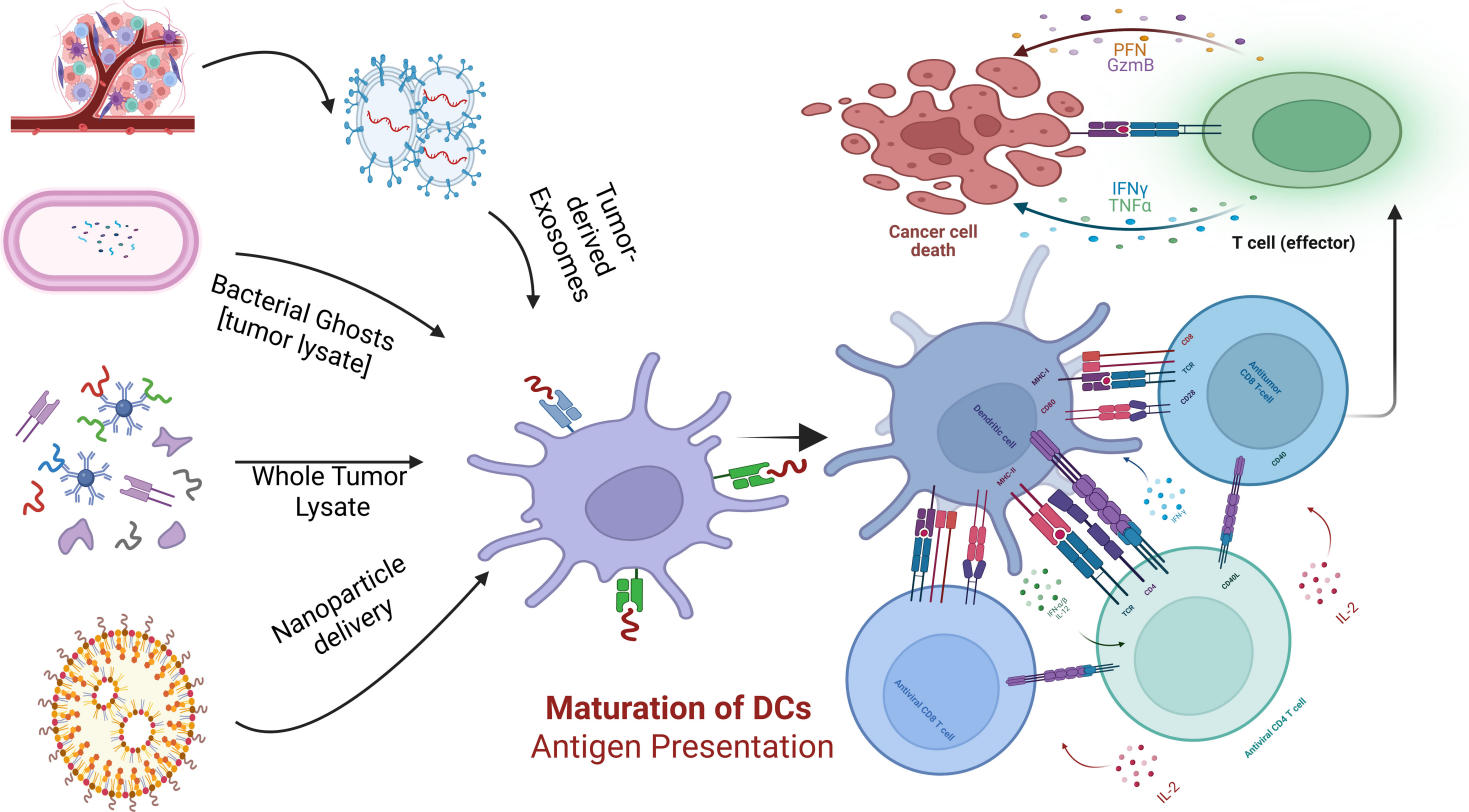
Targeting dendritic cells (DCs) with tumor lysates for cancer immunotherapy. Tumor lysates and their derivatives serve as rich sources of tumor-associated antigens to promote DC maturation and antigen presentation. Approaches include: (i) tumor-derived exosomes carrying antigenic cargo, (ii) bacterial ghosts providing adjuvant effects to overcome tumor-induced immunosuppression, (iii) whole tumor lysates supplying both defined and undefined antigens, and (iv) nanoparticle-based delivery platforms for efficient DC targeting. These strategies enhance the ability of DCs to present tumor antigens via MHC-I and MHC-II, upregulate co-stimulatory molecules (CD80, CD86, CD40), and activate CD8^+^ cytotoxic T cells and CD4^+^ helper T cells. The resulting effector T cell responses, characterized by IFN-γ, TNF-α, and cytotoxic mediators (perforin, granzyme B), drive antitumor immunity and promote cancer cell death.

DC-based vaccination initially generated considerable optimism; however, its therapeutic outcomes have often fallen short of expectations. Emerging evidence suggests that immunosuppressive mechanisms mediated by tumor-derived exosomes (TEX) may limit the clinical efficacy of such approaches (82). In studies using the mouse myeloid leukemia line WEHI3B and the renal cell carcinoma line RENCA, TEX served as an effective tumor antigen for DC loading. DC vaccination markedly prolonged the survival of WEHI3B-bearing mice, with DC-TEX showing greater efficacy than DC-lys. Importantly, even excess TEX did not interfere with the induction of immune responses (82). In addition, Bacterial Ghosts (BGs), empty envelopes derived from Gram-negative bacteria, have been explored as a platform for optimizing DC vaccine production. Retaining intact surface structures, BGs exhibit potent adjuvant properties that promote DC maturation. Importantly, BG-matured DCs were shown to withstand the immunosuppressive and pro-tolerogenic influences of various tumor cell lysates, including those from melanoma, renal carcinoma, and glioblastoma (83) (**Figure 2**). In a mouse breast cancer (MMTV-Ras) model, bone marrow-derived DCs pulsed with whole tumor lysate (TAA-DC) were characterized as a source of defined and undefined antigens. These antigen-loaded DCs exhibited a semi-mature to mature phenotype and upregulated genes associated with antigen presentation and T-cell priming. Such findings support the potential of DC-based antitumor therapies by demonstrating specific immune activation against breast cancer (84). Furthermore, Chitosan nanoparticles (CTS NPs) functionalized with mannose residues (Man-CTS NPs) were designed to achieve targeted delivery to DCs. These particles were subsequently loaded with tumor cell lysates (TCL) derived from B16 melanoma cells, generating Man-CTS-TCL NPs for both *in vitro* and *in vivo* evaluation. Vaccination with Man-CTS-TCL NPs significantly delayed tumor growth in mice, an effect largely attributable to the induction of cytotoxic T lymphocyte responses. In addition, the Man-CTS-TCL NP vaccine demonstrated therapeutic efficacy in established melanoma models (85).

A pilot clinical trial investigated a TuLy-DC vaccine, generated by culturing DCs with autologous tumor lysates (TuLy), in patients with metastatic renal cell carcinoma (mRCC). Tumor material was obtained via nephrectomy, and lysates were prepared by three freeze–thaw cycles. The trial confirmed the safety and feasibility of producing a DC-based vaccine for mRCC; however, clinical responses were limited, indicating that TuLy-DC vaccination alone may offer only modest therapeutic benefit (86). Similarly, another clinical trial evaluated DC-based immunotherapy and sunitinib in mRCC patients to enhance efficacy by inhibiting immunosuppressive cells. Sunitinib reduced MDSCs in the peripheral blood of five patients and Tregs in three patients. Tumor lysate–reactive CD4 or CD8 T cell responses were observed in five patients, four of whom showed decreased Tregs and/or MDSCs (87). A phase I/II study evaluated the safety, immunogenicity, and clinical activity of a vaccine composed of autologous DC loaded with an allogeneic tumor cell lysate in patients with advanced melanoma. The trial demonstrated that vaccination with DC pulsed with allogeneic melanoma lysate was feasible on a large scale and well tolerated in this patient cohort (88). The use of tumor lysates to pulse DCs has been investigated in preclinical and clinical trials. It is considered one of the safest approaches to enhance immunogenicity against solid tumors. However, only a few clinical studies have demonstrated meaningful success, and patient responses remain highly variable. This highlights the need to further optimize individualized and personalized DC-based therapies, emphasizing making such approaches more cost-effective and accessible. In addition, delivery platforms and pulsing strategies must be improved to maximize the therapeutic potential of tumor lysate–based DC vaccines in cancer.

### 4.3 Targeting DCs with nanoparticles

Various nanomaterial platforms, such as protein cage nanoparticles, biomimetic nanoparticles, and targeted multifunctional nanoparticles, have been engineered to improve the antigen-presenting capacity of DCs and to strengthen their ability to stimulate T cell responses (89). DCs encounter several obstacles that limit their clinical translation, including difficulties in controlling antigen dosage and their limited availability in peripheral blood. Although B cells have been considered an alternative platform for antigen presentation, their inherently poor nonspecific antigen uptake restricts their capacity to reliably prime T cells in a controlled manner (90). However, lipid-based NPs currently represent the most advanced platforms for cancer vaccine delivery, although polymer-based NPs are also gaining significant attention for their versatility and immunomodulatory potential (91). Therefore, phospholipid-conjugated antigens (L-Ags) and lipid–polymer hybrid nanoparticles (L/P-Ag NPs) have been developed as versatile delivery platforms to broaden the spectrum of antigen-presenting cells (APCs) engaged in T cell priming. Delivery of L-Ags, a process termed *depoting*, enables the successful loading of both MHC class I- and II-restricted antigens across diverse APC subsets in a tunable fashion, thereby promoting the activation of antigen-specific CD8^+^ and CD4^+^ T cells (90). Furthermore, integrating L-Ags and polymer-conjugated antigens (P-Ags) into nanoparticle systems can target distinct uptake pathways, enabling precise control over antigen presentation dynamics and shaping T cell responses. Importantly, while DCs effectively processed and presented antigen from both L- and P-Ag NPs, B cells were restricted to antigen derived from L-Ag NPs, resulting in distinct cytokine secretion patterns in coculture experiments (90).

In tumor-bearing mice, therapeutic administration of NP-CD40 significantly improved tumor control and prolonged survival. In this strategy, biodegradable poly(lactic-co-glycolic acid) nanoparticles were formulated to encapsulate a protein antigen, together with Pam3CSK4 and Poly(I: C), and were surface-coated with an agonistic αCD40 mAb (NP-CD40) (92). The clinical efficacy of cancer vaccines has been limited by tumor antigen heterogeneity and the dysfunctional cross-presentation capacity of tumor-infiltrating DCs (TIDCs). An *in situ* nanovaccine (TPOP), designed to regulate lipid metabolism and stimulate innate immunity, was developed to address these barriers. In murine colorectal cancer and melanoma models, intratumoral delivery of TPOP following doxorubicin pretreatment achieved strong therapeutic responses (93).

Similarly, a chimeric cross-linked polymosome (CCPS) formed by the self-assembly of the triblock copolymer PDMA has been developed as a multifunctional nanoplatform. CCPS is capable of co-encapsulating low-dose doxorubicin hydrochloride (DOX) to induce immunogenic cell death (ICD) and the photosensitizer HPPH to enable photodynamic therapy (PDT)–mediated reactive oxygen species (ROS) generation (94). *In vivo*, the all-in-one formulation (CCPS/HPPH/DOX) promoted the accumulation of mature DCs in tumor-draining lymph nodes and increased CD8^+^ T cell infiltration in tumor tissues, thereby inhibiting both primary and metastatic MC38 tumor growth after a single intravenous administration with low-dose DOX and HPPH (94).

**Figure 3:**
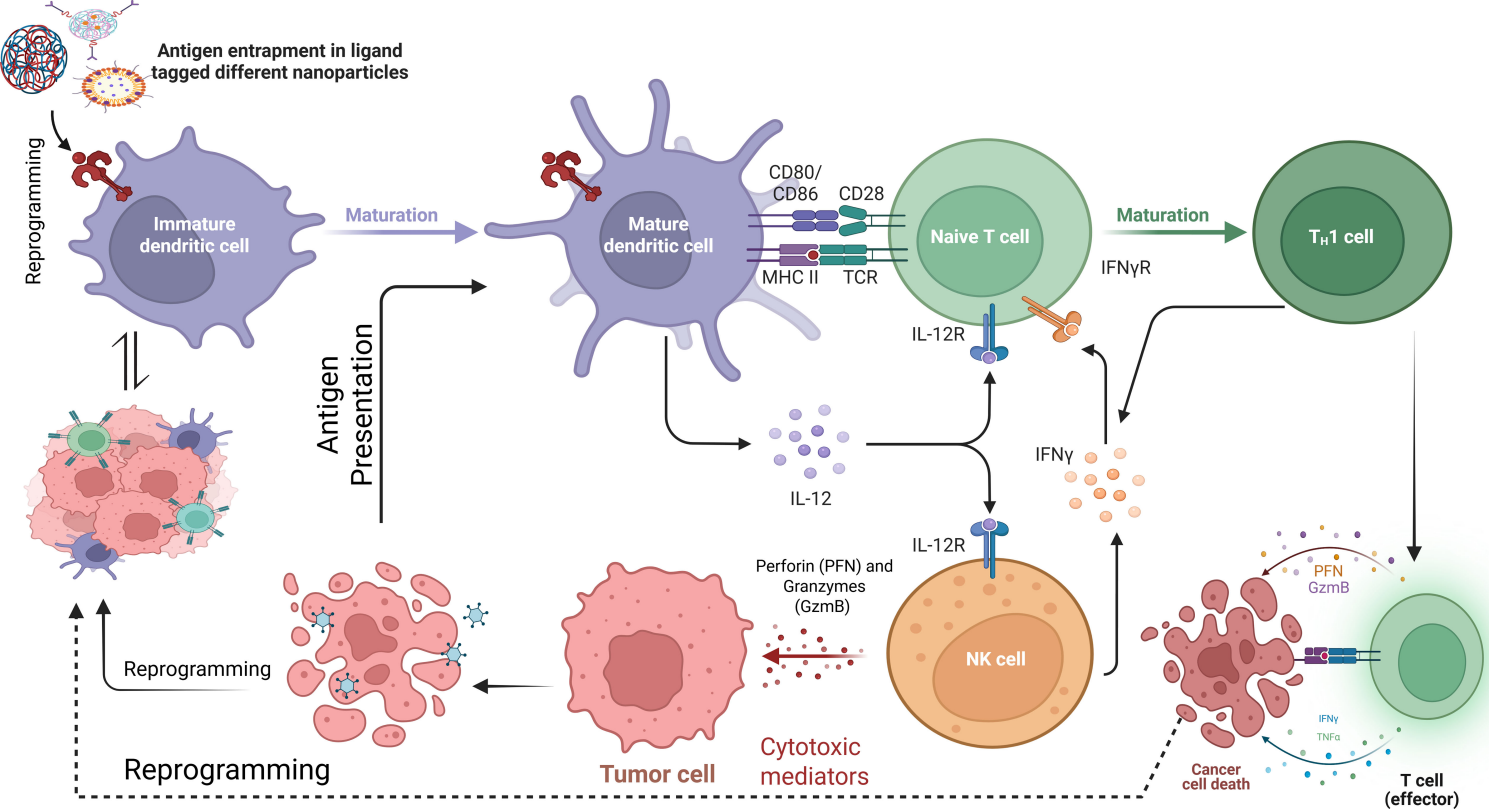
Nanoparticle-mediated reprogramming of dendritic cells and enhancement of antitumor immunity. Immature DCs on the TME internalize tumor-associated antigens entrapped in ligand-tagged nanoparticles, which drive their reprogramming and maturation into antigen-presenting mature DCs. These DCs activate naïve T cells through MHC–TCR interaction and CD80/CD86–CD28 costimulation, supported by IL-12 secretion. Activated T cells differentiate into Th1 and cytotoxic effector T cells, producing IFN-γ, TNF-α, perforin (PFN), and granzymes (GzmB) that mediate tumor cell killing. Concurrently, IL-12 enhances NK cell activation, which further contributes to tumor cytotoxicity. Reprogramming of dysfunctional DCs and the release of cytotoxic mediators converge to promote effective antitumor immunity and tumor cell elimination.

DC loading with NPL enhanced cytokine secretion by activating CD8^+^ T cells and promoting the expression of co-stimulatory surface molecules, consistent with effective antitumor immune responses [**Figure 3]**. By contrast, delivery of unmodified tumor lysate antigens predominantly generated a T cell phenotype associated with tolerization and exhaustion (95). Polymeric NPs have been explored for delivering the tumor-associated antigen Her2/neu to DCs to induce T cell responses via mucosal vaccination. When applied to the apical surface of an intestinal epithelium model, these NPs were efficiently transported across the epithelial barrier and became accessible to basolateral DCs. Head and neck squamous cell carcinoma (HNSCC) cell lines were used to prepare PLGA nanoparticles containing tumor-associated antigens (TAA). In patient samples, NP-mediated antigen delivery enhanced antitumor CD8^+^ T cell activity, reflected by a trend toward increased production of the immunostimulatory cytokine IFN-γ and a significant reduction in the immunoinhibitory cytokine IL-10 (96–98).

Functionalized alginate (ALG) nanoparticles (MAN-ALG/ALG=OVA NPs) for DC targeting, which enhanced antigen uptake and cytosolic release in BMDCs, thereby improving cross-presentation of OVA to B3Z T cell hybridoma *in vitro*. *In vivo*, subcutaneous administration of MAN-ALG/ALG=OVA NPs elicited strong cytotoxic T lymphocyte (CTL) responses and significantly suppressed E.G7 tumor growth in C57BL/6 mice (99). In addition, antigen-loaded upconversion nanoparticles (UCNPs) have been employed to label and activate DCs, enabling precise tracking after administration and induction of antigen-specific immune responses. DC vaccines pulsed with UCNPs elicited potent antigen-specific immunity, characterized by enhanced T cell proliferation, elevated interferon-γ production, and robust cytotoxic T lymphocyte (CTL) activity (100).

Evidence from two phase I clinical trials is particularly encouraging: prostate cancer patients immunized with DCs transfected with prostate-specific antigen mRNA, and renal cancer patients receiving DCs loaded with autologous tumor RNA, both showed vaccine-induced T-cell responses in the majority of cases (101). The application of autologous melanoma-derived mRNA enables targeting patient-specific tumor antigens, providing a personalized vaccination strategy. Robust protocols have been developed for mRNA transfection for producing clinical-grade DCs. A comprehensive preclinical study further validated this approach, demonstrating *in vitro* T-cell responses in all six advanced melanoma patients tested (102). Versatile cancer cell membrane (CCM)-coated calcium carbonate (CC) nanoparticles (MC) have been developed to generate *in situ* tumor-associated antigens (TAAs) for DC vaccination. MC/Dox/Ce6 treatment has effectively promoted TAA release and DC recruitment, initiating a downstream immune response cascade. Despite these advances, no nanoparticle-based DC therapy has yet reached the market for cancer treatment. Further optimization is required to improve delivery efficiency, reprogram immune responses within the TME, reduce or overcome immune checkpoint activity, and strengthen both DC pulsing and T cell activation to achieve effective cancer eradication.

### 4.4 Cytokine and chemokine therapy

Therapeutic approaches aimed at reversing DC tolerization within the TME are promising strategies, with strong potential for evaluation in future combination immunotherapy clinical trials (103). Cytokine-induced killer (CIK) cells exhibit potent cytolytic activity against a broad range of tumor cells *in vitro* and preclinical models. Their anti-tumor efficacy is further enhanced when activated through stimulation by DCs. Integrating DC-activated CIK cells with chemotherapy has improved therapeutic outcomes in malignant tumors, largely by upregulating cytokines that promote tumor cell elimination (104). Once underestimated, the role of GM-CSF in DC biology is now recognized as pivotal for maintaining DC homeostasis and driving their development under both steady-state and inflammatory conditions. GM-CSF exerts its effects by activating key intracellular signaling cascades, including JAK/STAT, MAPK, PI3K, and canonical NF-κB pathways. These signaling networks regulate transcription factors and downstream effector proteins that collectively govern the differentiation, survival, and proliferation of uncommitted hematopoietic progenitors and DC subset–specific precursors (105). Cytokines and chemokines offer a promising strategy to reprogram intrinsic immune cells, shifting them from an immunosuppressive phenotype toward a more immunostimulatory, antigen-presenting state (106). By restoring DC capacity via cytokines to process and present tumor antigens, these mediators can enhance anti-tumor T cell responses and significantly improve the effectiveness of cancer immunotherapy.

### 4.5 DC-based cellular therapies and vaccine clinical trials

DCs have been evaluated in cancer immunotherapy clinical trials for over 20 years. DC culture techniques, antigen-loading strategies, maturation signals, and administration routes have been extensively refined and diversified (107, 108). Despite low objective tumor response rates, typically under 15%, emerging evidence suggests that DC-based vaccines may enhance patient survival, highlighting the need for alternative endpoints to evaluate their clinical efficacy better (109). In prostate cancer patients receiving DC vaccines pulsed with PSM-derived peptides, clinical responses correlated with overall immunocompetence, including recall antigen reactivity and T-cell cytokine secretion, suggesting that assessing immune fitness may help predict and select responders to therapy (110). A meta-analysis demonstrates that DC-CIK immunotherapy offers important benefits in survival and response outcomes for solid tumors, with tolerable toxicity. However, the evidence is largely based on trials from China, and broader, high-quality studies are needed to confirm its clinical value (111). A phase II trial across six Indian centers demonstrated that autologous DC therapy in patients with refractory solid tumors was safe, achieving objective responses in nearly one-third of patients and encouraging survival outcomes (112). A study of 24 recurrent malignant glioma patients, autologous tumor lysate–pulsed dendritic cell therapy was safe, with modest clinical responses and improved survival in subsets receiving OK-432 maturation or combined intratumoral plus intradermal delivery (113). Similarly, in a randomized trial of metastatic melanoma patients, autologous DC vaccines loaded with tumor antigens showed superior survival outcomes to irradiated tumor cell vaccines, with a 2-year survival rate of 72% versus 31%. Both therapies were well tolerated (114).

Similarly, in 14 patients with stage IV solid tumors, intradermal administration of tumor lysate-pulsed DCs was safe, induced T-cell infiltration at vaccination sites, and generated measurable immune responses to both KLH and tumor antigens, with limited clinical responses observed (115). In addition, a trial of 17 malignant glioma patients, autologous DC-tumor vaccines were well tolerated aside from transient liver enzyme elevations, induced CD8^+^ T cell infiltration, and improved survival compared to historical controls, particularly in relapsed cases (116). In a curtailed randomized trial of 15 colon cancer patients with resected liver metastases, dendritic cell vaccines loaded with autologous tumor lysates showed a trend toward longer disease-free survival compared to observation, despite the small cohort size (117). In a phase I study of 10 HLA-A24 patients with advanced gastrointestinal or lung cancers, CEA652 peptide–pulsed DCs combined with interferon-α and TNF-α were safe and well tolerated, induced delayed-type hypersensitivity responses in a subset, and led to disease stabilization in 2 patients (118). In a trial of 31 advanced HCC patients, autologous tumor lysate–pulsed DC vaccination was safe, achieving partial responses in 12.9% and stable disease in 54.8%. Patients receiving pulsed plus booster therapy showed significantly improved one-year survival compared to pulsed therapy alone (119). In a phase I/II trial of 37 patients with advanced sarcomas, tumor lysate–pulsed DC therapy was safe and increased IFN-γ and IL-12. However, clinical benefit was limited, with only one partial response and a few cases of stable disease (115). Moreover, in a retrospective review of 1100 patients (2504 cycles) receiving DC-CIK therapy, most adverse events were linked to concurrent or prior chemotherapy, particularly hematological, gastrointestinal, and nutritional disorders. DC-CIK treatment alone was generally safe, with repeated cycles even reducing fatigue, anorexia, and anxiety (120).

Although DCs represent only a minor fraction of the TME, single-cell transcriptomic analyses highlight their heterogeneity and conserved functional states across cancers, underscoring their critical role in antitumor immunity (121). The phase I trial evaluated a DC vaccine engineered with an adenoviral HER2 construct in patients with metastatic HER2-positive cancers and high-risk bladder cancer. The vaccine was well tolerated, elicited strong cellular immune responses in most patients, and showed early clinical benefit in a subset, including complete and partial responses (122). In addition, intratumoral delivery of type 1 DCs, combined with ERBB2-targeted therapy before chemotherapy, was safe and immunogenic, with promising clinical responses in a phase 1 trial in ERBB2-positive breast cancer. Importantly, dose escalation influenced T-cell dynamics, with several patients achieving pathologic complete responses (123).

Beyond single-agent DC vaccination, combination strategies integrating DC therapy with chemotherapy, radiotherapy, immune checkpoint inhibitors, and cell-based therapies have been evaluated in clinical settings. Integrating DC vaccines with additional cancer treatments, including chemotherapy and monoclonal antibodies, can improve antitumor efficacy and overall therapeutic outcomes (124). Significant strategies have focused on combining DC therapy with chemotherapy, radiotherapy, or immune checkpoint inhibitors to overcome tumor-mediated immunosuppression and maximize the efficacy of DC-based therapies (125). Achieving robust clinical efficacy with DC immunotherapy remains a major hurdle. An emerging strategy involves combining DC-based vaccines with immune checkpoint inhibitors (ICIs), including antibodies against CTLA-4 and PD-1. Such combinations have demonstrated the potential to amplify T cell responses and improve therapeutic outcomes. Importantly, these synergistic approaches can convert immunologically “cold” tumors into “hot” tumors, thereby enhancing the overall effectiveness of ICIs (126).

AcTakines are engineered cytokines directed to DCs that elicit strong antitumor responses in murine melanoma, breast cancer, and lymphoma models, as well as in humanized mouse lymphoma models, without detectable toxicity. Combination with checkpoint inhibitors, chemotherapy, or low-dose TNF enhances tumor regression and establishes long-term antitumor immunity, while maintaining a favorable safety profile (127, 128). In a phase I/II study, ten cancer patients received autologous dendritic cell vaccines combined with low-dose IL-2. These were well tolerated and elicited antigen-specific immune responses, including enhanced lymphocyte proliferation, IFN-γ secretion, and NK activity. Clinical benefit correlated with a combination of immune activation and reduction of inhibitory mechanisms, highlighting the potential of DC vaccines to induce targeted antitumor immunity (129).

Combination strategies integrating DCs with other immunotherapeutic agents have shown promising results. A meta-analysis demonstrated that DC-CIK immunotherapy offers meaningful survival and response benefits in solid tumors with tolerable toxicity, though the evidence remains largely based in China, and broader studies are needed (130). Supporting this, a retrospective review of 1100 patients receiving DC-CIK therapy confirmed its safety profile, with adverse events largely attributable to concurrent chemotherapy, and repeated cycles even reducing fatigue, anorexia, and anxiety (120, 122). Beyond CIK combinations, intratumoral delivery of type 1 DCs combined with ERBB2-targeted therapy prior to chemotherapy in ERBB2-positive breast cancer demonstrated safety, immunogenicity, and promising clinical responses, with dose escalation influencing T-cell dynamics and several patients achieving pathologic complete responses (123).

Combining DC administration with paclitaxel chemotherapy in murine mammary adenocarcinoma induced robust antigen-specific CD8 and CD4 T-cell responses, but only when DCs were injected into the tumor site. This combinatorial approach generated significant antitumor effects, demonstrating the potential of DC-based immunotherapy even under immunosuppressive conditions (112). Cytokine-induced killer (CIK) cells, a CD3^+^CD56^+^ lymphocyte subset, exhibit potent antitumor activity. Combining CIKs with DCs further enhances antitumor efficacy, offering a promising adoptive cellular immunotherapy strategy (131). Combining DC and cytokine-induced killer cell immunotherapy with conventional chemotherapy in late-stage NSCLC is safe and tolerable and prolongs time to disease progression. This chemoimmunotherapy approach can improve patient outcomes compared to chemotherapy alone (132). Combination strategies integrating chemotherapy, immune checkpoint inhibitors, monoclonal antibodies, cytokines, or cell-based therapies such as CAR-T must be optimized for individual patients due to tumor heterogeneity. Tailoring these approaches could maximize antitumor efficacy, minimize adverse effects, and improve therapeutic outcomes, particularly in solid tumors. Although most clinical trials with DC vaccines have demonstrated encouraging outcomes, the overall success rate remains modest at approximately 15% (12). This underscores the need for a deeper understanding of how DC immunotherapy can be optimized to enhance cancer treatment, particularly by improving antigen presentation and functional reprogramming of DCs. Despite limited efficacy, DC-based therapies have consistently shown excellent safety and tolerability compared with other cancer immunotherapies.

## 5 Challenges and opportunities

DCs are central to cancer immunotherapy because they can trigger strong, tumor-specific immune responses, especially during early tumor development. However, as tumors advance, DCs can adopt a regulatory phenotype that promotes an immunosuppressive microenvironment and supports tumor progression. Modulating DC phenotype may therefore hold promise for enhancing anti-tumor immunity, though the complexity of tumor immune evasion presents a significant challenge (133). Although DC-based vaccines aim to bypass impaired DC maturation, obstacles such as inefficient migration to T-cell priming sites and the suppressive TME have limited their ability to overcome tumor-induced tolerance. Importantly, a successful trial with autologous tumor lysate–pulsed DC vaccination in recurrent ovarian cancer is a promising exception (133, 134).

Although DC-based cancer vaccines have shown the capacity to enhance host CD8 T cell–mediated antitumor activity, their clinical impact has thus far been limited, with the important exception of the FDA-approved Provenge. Recent evidence highlights the pivotal role of type 1 conventional DCs (cDC1s) in cross-priming tumor-specific CD8 T cells and dictating the efficacy of cancer immunotherapies, including immune checkpoint blockade. In parallel, encouraging outcomes from neoantigen-based cancer vaccines underscore the urgent need to further optimize DC-based strategies, either as stand-alone approaches or in synergistic combination with other immunotherapies (135). Immunotherapy has sought to harness the unique capacity of DCs to present antigens for therapeutic vaccination in patients with advanced cancers. Since the landmark clinical trial in melanoma in 1995, DC–based vaccines have been investigated extensively across multiple phase I and II studies. Although important progress has been achieved, particularly in prostate cancer, significant obstacles must be overcome to enhance their clinical efficacy in future trials (136).

DCs can be generated *in vitro* from multiple cellular sources, including bone marrow, cord, and peripheral blood. Although culture protocols vary considerably, most rely on GM-CSF combined with IL-4 and/or TNF-α to drive DC differentiation. Supplementation with additional cytokines such as stem cell factor (SCF) or Flt3 ligand has markedly improved DC yield. Distinct DC subsets can be derived, and culture conditions strongly influence their maturation state, phenotypic profile, and antigen-capturing capacity (137). Over the past several years, various DC-based approaches, particularly those employing *ex vivo–*generated monocyte-derived DCs, have been evaluated in preclinical and clinical multiple myeloma studies (MM). Nevertheless, these strategies have yielded only limited long-term clinical benefit, likely due to the suboptimal functionality of monocyte-derived DCs combined with the profoundly immunosuppressive bone marrow microenvironment (138).

Numerous studies have investigated DC–based vaccination strategies using tumor-derived antigens to immunize B-cell malignancies, though clinical outcomes have been modest. To identify more effective antigens for DC-based vaccination in MM, comparative studies were conducted using DCs pulsed with either the idiotype (Id) protein or tumor lysate in the 5TGM1 murine myeloma model. Both approaches demonstrated robust protective efficacy, delaying tumor onset, inhibiting progression, and even inducing regression in established disease. Moreover, vaccinated mice that achieved long-term survival were resistant to subsequent tumor rechallenge, underscoring the therapeutic potential of optimized DC-based vaccines in myeloma (139). A major contributing factor is reliance on *ex vivo*–generated monocyte-derived DCs (MoDCs), which often fail to fully recapitulate the functional properties of naturally occurring DC subsets. Recent advances highlighting the pivotal role of the rare conventional type 1 DC (cDC1) subset in initiating cytotoxic CD8^+^ T cell responses have reshaped the field. This has fostered a paradigm shift toward exploiting cDC1 biology as a more effective strategy to potentiate anti-tumor immunity and improve therapeutic outcomes in cancer patients (140, 141).

The safety profile of DC vaccination and its capacity to elicit antitumor immune responses are now well established. Nevertheless, despite occasional reports of durable clinical benefit in individual patients, DC vaccines have yet to achieve their full therapeutic potential. A key limitation has been the scarcity of large, rigorously conducted phase II and III trials (142). Progress toward meaningful multicenter trials will require standardized protocols for DC vaccine production, which is now increasingly attainable. Moreover, enhancing the efficacy of DC-based immunotherapy may depend on its rational integration with other treatment modalities (142, 143).

## 6 Conclusion

DC-based reprogramming strategies to restore DC functionality in cancer represent a promising avenue to overcome tumor-induced immune evasion. Nonetheless, the ongoing disconnect between strong preclinical findings and modest clinical results suggests that existing strategies have not yet fully accounted for the complexity of the human tumor microenvironment. By rescuing dysfunctional DCs from their immunosuppressive state and enhancing their antigen-presenting capacity, it may be possible to improve CD8^+^ T cell activation. Integrating novel delivery systems, such as nanoparticles (including PLGA, polymeric, selenium-based, and other platforms), with DC-based therapies offers opportunities to augment therapeutic efficacy and precision.

DC therapy (DCT) has advanced through various approaches in generation, administration, and optimization, yet multiple challenges remain. Critically, whether reprogrammed DCs can retain their immunostimulatory phenotype long enough to sustain meaningful antitumor responses *in vivo* in the presence of persistent tumor-derived suppressive signals remains a fundamental question. It is important to recognize that epigenetic reprogramming, cytokine and chemokine modulation, and TLR-mediated activation represent mechanistically distinct intervention points supported by largely non-overlapping experimental evidence; therefore, direct comparative studies under physiologically relevant TME conditions are needed to establish which strategies offer the greatest translational potential, particularly how dysregulated cytokine and chemokine signaling and tumor-induced immunosuppressive pathways impair DC functionality and antigen presentation. Addressing these barriers is essential to enhancing therapeutic efficacy. Furthermore, combining DC vaccines with immune checkpoint inhibitors, chemotherapy, or radiotherapy may help bridge this translational gap, provided that future studies prioritize understanding the optimal sequencing and mechanistic basis of such combinations, rather than assuming their effects are simply additive.

Furthermore, mechanistic insights into the roles of epigenetic reprogramming, cytokine and chemokine signaling, tumor-derived suppressive pathways, and TLR signaling could help in developing more robust strategies to convert dysfunctional DCs back into fully functional immunostimulatory cells. Advances in single-cell sequencing, spatial transcriptomics, and proteomics will be instrumental in identifying key biomarkers and metabolic pathways that could be targeted to reprogram DCs, ultimately unlocking the full potential of DC-based immunotherapy.

## References

[1] Ma H Fang W Li Q Wang Y Hou SX . Arf1 ablation in colorectal cancer cells activates a super signal complex in DC to enhance anti-tumor immunity. Adv Sci (Weinheim Baden-Wurttemberg Ger). (2023) 10. doi: 10.1002/ADVS.202305089 37786300 PMC10646219

[2] Wang YH Ito T Wang YH Homey B Watanabe N Martin R . Maintenance and polarization of human TH2 central memory T cells by thymic stromal lymphopoietin-activated dendritic cells. Immunity. (2006) 24:827–38. doi: 10.1016/j.immuni.2006.03.019 16782037

[3] Macri C Pang ES Patton T O’Keeffe M . Dendritic cell subsets. Semin Cell Dev Biol. (2018) 84:11–21. doi: 10.1016/j.semcdb.2017.12.009 29246859

[4] Kvedaraite E Ginhoux F . Human dendritic cells in cancer. Sci Immunol. (2022) 7. doi: 10.1126/SCIIMMUNOL.ABM9409 35363544

[5] Del Prete A Salvi V Soriani A Laffranchi M Sozio F Bosisio D . Dendritic cell subsets in cancer immunity and tumor antigen sensing. Cell Mol Immunol. (2023) 20:432–47. doi: 10.1038/S41423-023-00990-6 36949244 PMC10203372

[6] Schlitzer A Sivakamasundari V Chen J Sumatoh HB Schreuder J Lum J . Identification of cDC1- and cDC2-committed DC progenitors reveals early lineage priming at the common DC progenitor stage in the bone marrow. Nat Immunol. (2015) 16:718–28. doi: 10.1038/NI.3200 26054720

[7] Backer RA Diener N Clausen BE . Langerin+CD8+ dendritic cells in the splenic marginal zone: Not so marginal after all. Front Immunol. (2019) 10:741. doi: 10.3389/FIMMU.2019.00741 31031751 PMC6474365

[8] MacNabb BW Tumuluru S Chen X Godfrey J Kasal DN Yu J . Dendritic cells can prime anti-tumor CD8+ T cell responses through major histocompatibility complex cross-dressing. Immunity. (2022) 55:982–997.e8. doi: 10.1016/j.immuni.2022.04.016 35617964 PMC9883788

[9] Joffre OP Segura E Savina A Amigorena S . Cross-presentation by dendritic cells. Nat Rev Immunol. (2012) 12:557–69. doi: 10.1038/NRI3254 22790179

[10] Schiavoni G Mattei F Gabriele L . Type I interferons as stimulators of DC-mediated cross-priming: Impact on anti-tumor response. Front Immunol. (2013) 4. doi: 10.3389/FIMMU.2013.00483 24400008 PMC3872318

[11] Fu C Zhou L Mi QS Jiang A . Plasmacytoid dendritic cells and cancer immunotherapy. Cells. (2022) 11. doi: 10.3390/CELLS11020222 35053338 PMC8773673

[12] Fu C Zhou L Mi QS Jiang A . DC-based vaccines for cancer immunotherapy. Vaccines. (2020) 8(4):706. doi: 10.3390/VACCINES8040706 33255895 PMC7712957

[13] Conrad C Gilliet M . Plasmacytoid dendritic cells and regulatory T cells in the tumor microenvironment: A dangerous liaison. Oncoimmunology. (2013) 2:e23887. doi: 10.4161/ONCI.23887 23762788 PMC3667894

[14] Fu C Peng P Loschko J Feng L Pham P Cui W . Plasmacytoid dendritic cells cross-prime naive CD8 T cells by transferring antigen to conventional dendritic cells through exosomes. Proc Natl Acad Sci USA. (2020) 117:23730–41. doi: 10.1073/PNAS.2002345117 32879009 PMC7519282

[15] Roberts EW Broz ML Binnewies M Headley MB Nelson AE Wolf DM . Critical role for CD103+/CD141+ dendritic cells bearing CCR7 for tumor antigen trafficking and priming of T cell immunity in melanoma. Cancer Cell. (2016) 30:324–36. doi: 10.1016/j.ccell.2016.06.003 27424807 PMC5374862

[16] Nierkens S Den Brok MH Garcia Z Togher S Wagenaars J Wassink M . Immune adjuvant efficacy of CpG oligonucleotide in cancer treatment is founded specifically upon TLR9 function in plasmacytoid dendritic cells. Cancer Res. (2011) 71:6428–37. doi: 10.1158/0008-5472.CAN-11-2154 21788345 PMC3653311

[17] Ma Y Shurin GV Peiyuan Z Shurin MR . Dendritic cells in the cancer microenvironment. J Cancer. (2013) 4:36–44. doi: 10.7150/JCA.5046 23386903 PMC3564245

[18] Steinman RM Hawiger D Nussenzweig MC . Tolerogenic dendritic cells. Annu Rev Immunol. (2003) 21:685–711. doi: 10.1146/ANNUREV.IMMUNOL.21.120601.141040 12615891

[19] Salmon H Idoyaga J Rahman A Leboeuf M Remark R Jordan S . Expansion and activation of CD103+ dendritic cell progenitors at the tumor site enhances tumor responses to therapeutic PD-L1 and BRAF inhibition. Immunity. (2016) 44:924–38. doi: 10.1016/j.immuni.2016.03.012 27096321 PMC4980762

[20] Garris CS Luke JJ . Dendritic cells, the T-cell-inflamed tumor microenvironment, and immunotherapy treatment response. Clin Cancer Res. (2020) 26:3901–7. doi: 10.1158/1078-0432.CCR-19-1321 32332013 PMC7607412

[21] Fu C Jiang A . Dendritic cells and CD8 T cell immunity in tumor microenvironment. Front Immunol. (2018) 9:3059. doi: 10.3389/FIMMU.2018.03059 30619378 PMC6306491

[22] Xiao Z Wang R Wang X Yang H Dong J He X . Impaired function of dendritic cells within the tumor microenvironment. Front Immunol. (2023) 14. doi: 10.3389/FIMMU.2023.1213629 37441069 PMC10333501

[23] Bakhshi P Nourizadeh M Sharifi L Nowroozi MR Mohsenzadegan M Farajollahi MM . Impaired monocyte‐derived dendritic cell phenotype in prostate cancer patients: A phenotypic comparison with healthy donors. Cancer Rep. (2024) 7:e1996. doi: 10.1002/CNR2.1996 38351552 PMC10864738

[24] Ma Y Shurin GV Gutkin DW Shurin MR . Tumor associated regulatory dendritic cells. Semin Cancer Biol. (2012) 22:298. doi: 10.1016/J.SEMCANCER.2012.02.010 22414911 PMC3373995

[25] Warrick KA Vallez CN Meibers HE Pasare C . Bidirectional communication between the innate and adaptive immune systems. Annu Rev Immunol. (2025) 43:489. doi: 10.1146/ANNUREV-IMMUNOL-083122-040624 40279312 PMC12120936

[26] Marciscano AE Anandasabapathy N . The role of dendritic cells in cancer and anti-tumor immunity. Semin Immunol. (2021) 52:101481. doi: 10.1016/J.SMIM.2021.101481 34023170 PMC8545750

[27] Belderbos RA Aerts JGJV Vroman H . Enhancing dendritic cell therapy in solid tumors with immunomodulating conventional treatment. Mol Ther Oncolytics. (2019) 13:67–81. doi: 10.1016/j.omto.2019.03.007 31020037 PMC6475716

[28] Nace G Evankovich J Eid R Tsung A . Dendritic cells and damage-associated molecular patterns: endogenous danger signals linking innate and adaptive immunity. J Innate Immun. (2012) 4:6–15. doi: 10.1159/000334245 22086146

[29] Sabado RL Balan S Bhardwaj N . Dendritic cell-based immunotherapy. Cell Res. (2017) 27:74–95. doi: 10.1038/CR.2016.157 28025976 PMC5223236

[30] Mempel TR Henrickson SE Von Andrian UH . T-cell priming by dendritic cells in lymph nodes occurs in three distinct phases. Nature. (2004) 427:154–9. doi: 10.1038/NATURE02238 14712275

[31] Nurieva R Thomas S Nguyen T Martin-Orozco N Wang Y Kaja MK . T-cell tolerance or function is determined by combinatorial costimulatory signals. EMBO J. (2006) 25:2623–33. doi: 10.1038/SJ.EMBOJ.7601146 16724117 PMC1478197

[32] Lutz MB Schuler G . Immature, semi-mature and fully mature dendritic cells: Which signals induce tolerance or immunity? Trends Immunol. (2002) 23:445–9. doi: 10.1016/S1471-4906(02)02281-0 12200066

[33] Chen DS Mellman I . Oncology meets immunology: The cancer-immunity cycle. Immunity. (2013) 39:1–10. doi: 10.1016/j.immuni.2013.07.012 23890059

[34] Larmonier N Marron M Zeng Y Cantrell J Romanoski A Sepassi M . Tumor-derived CD4(+)CD25(+) regulatory T cell suppression of dendritic cell function involves TGF-beta and IL-10. Cancer Immunol Immunother. (2007) 56:48–59. doi: 10.1007/S00262-006-0160-8 16612596 PMC11030031

[35] Ohtani H Terashima T Sato E . Immune cell expression of TGFβ1 in cancer with lymphoid stroma: dendritic cell and regulatory T cell contact. Virchows Arch. (2018) 472:1021–8. doi: 10.1007/S00428-018-2336-Y 29594353 PMC5999139

[36] Dumitriu IE Dunbar DR Howie SE Sethi T Gregory CD . Human dendritic cells produce TGF-beta 1 under the influence of lung carcinoma cells and prime the differentiation of CD4+CD25+Foxp3+ regulatory T cells. J Immunol. (2009) 182:2795–807. doi: 10.4049/JIMMUNOL.0712671 19234174

[37] Trebska-McGowan K Chaib M Alvarez MA Kansal R Pingili AK Shibata D . TGF-β alters the proportion of infiltrating immune cells in a pancreatic ductal adenocarcinoma. J Gastrointest Surg. (2021) 26:113–21. doi: 10.1007/S11605-021-05087-X 34260016 PMC12289350

[38] Tang M Diao J Gu H Khatri I Zhao J Cattral MS . Toll-like receptor 2 activation promotes tumor dendritic cell dysfunction by regulating IL-6 and IL-10 receptor signaling. Cell Rep. (2015) 13:2851–64. doi: 10.1016/j.celrep.2015.11.053 26711349

[39] Gabrilovich D . Mechanisms and functional significance of tumour-induced dendritic-cell defects. Nat Rev Immunol. (2004) 4:941–52. doi: 10.1038/NRI1498 15573129

[40] Yu H Kortylewski M Pardoll D . Crosstalk between cancer and immune cells: role of STAT3 in the tumour microenvironment. Nat Rev Immunol. (2007) 7:41–51. doi: 10.1038/NRI1995 17186030

[41] Ouyang FZ Wu RQ Wei Y Liu RX Yang D Xiao X . Dendritic cell-elicited B-cell activation fosters immune privilege via IL-10 signals in hepatocellular carcinoma. Nat Commun. (2016) 7. doi: 10.1038/NCOMMS13453 27853178 PMC5118541

[42] Steinbrink K Wölfl M Jonuleit H Knop J Enk AH . Induction of tolerance by IL-10-treated dendritic cells. J Immunol. (1997) 159:4772–80. doi: 10.4049/jimmunol.159.10.4772 9366401

[43] Han Z Dong Y Lu J Yang F Zheng Y Yang H . Role of hypoxia in inhibiting dendritic cells by VEGF signaling in tumor microenvironments: mechanism and application. Am J Cancer Res. (2021) 11:3777. 34522449 PMC8414384

[44] Mimura K Kono K Takahashi A Kawaguchi Y Fujii H . Vascular endothelial growth factor inhibits the function of human mature dendritic cells mediated by VEGF receptor-2. Cancer Immunol Immunother. (2007) 56:761–70. doi: 10.1007/S00262-006-0234-7 17086423 PMC11030780

[45] Gulubova M Ivanova K Ananiev J Gerenova J Zdraveski A Stoyanov H . VEGF expression, microvessel density and dendritic cell decrease in thyroid cancer. Biotechnol Biotechnol Equip. (2014) 28:508–17. doi: 10.1080/13102818.2014.909151 26019537 PMC4433839

[46] Della Porta M Danova M Rigolin GM Brugnatelli S Rovati B Tronconi C . Dendritic cells and vascular endothelial growth factor in colorectal cancer: correlations with clinicobiological findings. Oncology. (2005) 68:276–84. doi: 10.1159/000086784 16015045

[47] Troise D Infante B Mercuri S Catalano V Ranieri E Stallone G . Dendritic cells: A bridge between tolerance induction and cancer development in transplantation setting. Biomedicines. (2024) 12:1240. doi: 10.3390/BIOMEDICINES12061240 38927447 PMC11200833

[48] Paardekooper LM Vos W Van Den Bogaart G . Oxygen in the tumor microenvironment: effects on dendritic cell function. Oncotarget. (2019) 10:883–96. doi: 10.18632/ONCOTARGET.26608 30783517 PMC6368231

[49] Mancino A Schioppa T Larghi P Pasqualini F Nebuloni M Chen IH . Divergent effects of hypoxia on dendritic cell functions. Blood. (2008) 112:3723–34. doi: 10.1182/BLOOD-2008-02-142091 18694997

[50] Suthen S Lim CJ Nguyen PHD Dutertre CA Lai HLH Wasser M . Hypoxia-driven immunosuppression by Treg and type-2 conventional dendritic cells in HCC. Hepatology. (2022) 76:1329–44. doi: 10.1002/HEP.32419 35184329

[51] Kierans SJ Taylor CT . Regulation of glycolysis by the hypoxia-inducible factor (HIF): implications for cellular physiology. J Physiol. (2021) 599:23–37. doi: 10.1113/JP280572 33006160

[52] Shaikh SR Edidin M . Polyunsaturated fatty acids, membrane organization, T cells, and antigen presentation. Am J Clin Nutr. (2006) 84:1277–89. doi: 10.1093/ajcn/84.6.1277 17158407

[53] Herber DL Cao W Nefedova Y Novitskiy SV Nagaraj S Tyurin VA . Lipid accumulation and dendritic cell dysfunction in cancer. Nat Med. (2010) 16:880–6. doi: 10.1038/NM.2172 20622859 PMC2917488

[54] Peng X He Y Huang J Tao Y Liu S . Metabolism of dendritic cells in tumor microenvironment: For immunotherapy. Front Immunol. (2021) 12. doi: 10.3389/FIMMU.2021.613492 33732237 PMC7959811

[55] Krawczyk CM Holowka T Sun J Blagih J Amiel E DeBerardinis RJ . Toll-like receptor-induced changes in glycolytic metabolism regulate dendritic cell activation. Blood. (2010) 115:4742–9. doi: 10.1182/BLOOD-2009-10-249540 20351312 PMC2890190

[56] Zhang B Zhao L Li H Wang N Wang X Shang L . Glycolysis in the tumor microenvironment shapes dendritic cell function and antitumor immunity. Front Immunol. (2026) 17:1744671. doi: 10.3389/FIMMU.2026.1744671 41737218 PMC12926433

[57] Silva Barcelos EC Ricciuti D Mondanelli G Gargaro M . Rewriting the dendritic cell code in cancer-from subset identity to immunotherapeutic design. FEBS Lett. (2025) 599:2060–83. doi: 10.1002/1873-3468.70108 40667699 PMC12302065

[58] Henriques-Oliveira L Altman AR Kurochkin I Ascic E Halitzki E Matei A-M . Anchored screening identifies transcription factor blueprints underlying dendritic cell diversity and subset-specific anti-tumor immunity. Immunity. (2025) 58(10):2419-38.e13. doi: 10.1016/J.IMMUNI.2025.08.001 40885192

[59] Li R Fang F Jiang M Wang C Ma J Kang W . STAT3 and NF-κB are simultaneously suppressed in dendritic cells in lung cancer. Sci Rep. (2017) 7. doi: 10.1038/SREP45395 28350008 PMC5368983

[60] Tas SW de Jong EC Hajji N May MJ Ghosh S Vervoordeldonk MJ . Selective inhibition of NF-kappaB in dendritic cells by the NEMO-binding domain peptide blocks maturation and prevents T cell proliferation and polarization. Eur J Immunol. (2005) 35:1164–74. doi: 10.1002/EJI.200425956 15770694

[61] Pfeiffer IA Hoyer S Gerer KF Voll RE Knippertz I Gückel E . Triggering of NF-κB in cytokine-matured human DCs generates superior DCs for T-cell priming in cancer immunotherapy. Eur J Immunol. (2014) 44:3413–28. doi: 10.1002/EJI.201344417 25100611

[62] Morante-Palacios O Godoy-Tena G Calafell-Segura J Ciudad L Martínez-Cáceres EM Sardina JL . Vitamin C enhances NF-κB-driven epigenomic reprogramming and boosts the immunogenic properties of dendritic cells. Nucleic Acids Res. (2022) 50:10981–94. doi: 10.1093/NAR/GKAC941 36305821 PMC9638940

[63] Zimmermannova O Ferreira AG Ascic E Santiago MV Kurochkin I Hansen M . Restoring tumor immunogenicity with dendritic cell reprogramming. Sci Immunol. (2023) 8. doi: 10.1126/SCIIMMUNOL.ADD4817 37418548 PMC7614848

[64] Poulin LF Reyal Y Uronen-Hansson H Schraml BU Sancho D Murphy KM . DNGR-1 is a specific and universal marker of mouse and human Batf3-dependent dendritic cells in lymphoid and nonlymphoid tissues. Blood. (2012) 119:6052–62. doi: 10.1182/BLOOD-2012-01-406967 22442345

[65] Barry KC Hsu J Broz ML Cueto FJ Binnewies M Combes AJ . A natural killer-dendritic cell axis defines checkpoint therapy-responsive tumor microenvironments. Nat Med. (2018) 24:1178–91. doi: 10.1038/S41591-018-0085-8 29942093 PMC6475503

[66] Ascic E Åkerström F Nair MS Rosa A Kurochkin I Zimmermannova O . *In vivo* dendritic cell reprogramming for cancer immunotherapy. Science. (2024) 386. doi: 10.1126/SCIENCE.ADN9083 39236156 PMC7616765

[67] Rosa FF Pires CF Kurochkin I Ferreira AG Gomes AM Palma LG . Direct reprogramming of fibroblasts into antigen-presenting dendritic cells. Sci Immunol. (2018) 3. doi: 10.1126/SCIIMMUNOL.AAU4292 30530727

[68] Yang Y Wang Y . Role of epigenetic regulation in plasticity of tumor immune microenvironment. Front Immunol. (2021) 12. doi: 10.3389/FIMMU.2021.640369 33868269 PMC8051582

[69] Doñas C Carrasco M Fritz M Prado C Tejón G Osorio-Barrios F . The histone demethylase inhibitor GSK-J4 limits inflammation through the induction of a tolerogenic phenotype on DCs. J Autoimmun. (2016) 75:105–17. doi: 10.1016/j.jaut.2016.07.011 27528513

[70] Godoy-Tena G Ballestar E . Epigenetics of dendritic cells in tumor immunology. Cancers (Basel). (2022) 14. doi: 10.3390/CANCERS14051179 35267487 PMC8909611

[71] Zhou Z Chen H Xie R Wang H Li S Xu Q . Epigenetically modulated FOXM1 suppresses dendritic cell maturation in pancreatic cancer and colon cancer. Mol Oncol. (2019) 13:873–93. doi: 10.1002/1878-0261.12443 30628173 PMC6441919

[72] Dai E Zhu Z Wahed S Qu Z Storkus WJ Guo ZS . Epigenetic modulation of antitumor immunity for improved cancer immunotherapy. Mol Cancer. (2021) 20. doi: 10.1186/S12943-021-01464-X 34930302 PMC8691037

[73] Galluzzi L Buqué A Kepp O Zitvogel L Kroemer G . Immunogenic cell death in cancer and infectious disease. Nat Rev Immunol. (2017) 17:97–111. doi: 10.1038/NRI.2016.107 27748397

[74] Jiang M-C Ni J-J Cui W-Y Wang B-Y Zhuo W . Emerging roles of lncRNA in cancer and therapeutic opportunities. Am J Cancer Res. (2019) 9:1354. 31392074 PMC6682721

[75] Cheng CJ Bahal R Babar IA Pincus Z Barrera F Liu C . MicroRNA silencing for cancer therapy targeted to the tumour microenvironment. Nature. (2015) 518:107–10. doi: 10.1038/NATURE13905 25409146 PMC4367962

[76] Palucka K Banchereau J . Dendritic-cell-based therapeutic cancer vaccines. Immunity. (2013) 39:38–48. doi: 10.1016/j.immuni.2013.07.004 23890062 PMC3788678

[77] Cranmer LD Trevor KT Hersh EM . Clinical applications of dendritic cell vaccination in the treatment of cancer. Cancer Immunol Immunother. (2004) 53:275–306. doi: 10.1007/S00262-003-0432-5 14648069 PMC11032969

[78] Guo Z Yuan Y Chen C Lin J Ma Q Liu G . Durable complete response to neoantigen-loaded dendritic-cell vaccine following anti-PD-1 therapy in metastatic gastric cancer. NPJ Precis Oncol. (2022) 6. doi: 10.1038/S41698-022-00279-3 35661819 PMC9166775

[79] Papakonstantinou M Chatzikomnitsa P Gkaitatzi AD Myriskou A Giakoustidis A Giakoustidis D . The efficacy of neoantigen-loaded dendritic cell vaccine immunotherapy in non-metastatic gastric cancer. Med Sci (Basel Switzerland). (2025) 13. doi: 10.3390/MEDSCI13030090 40700119 PMC12285992

[80] Li J Li J Peng Y Du Y Yang Z Qi X . Dendritic cell derived exosomes loaded neoantigens for personalized cancer immunotherapies. J Control Release. (2023) 353:423–33. doi: 10.1016/j.jconrel.2022.11.053 36470333

[81] Dhar R Sonar S Das A Tajul Akmal NAS Hawa Jasni A RMT Balasubramaniam V . Dendritic cell-derived exosomes: Next generation of cancer immunotherapy. Biomedicines. (2025) 13:2497. doi: 10.3390/BIOMEDICINES13102497 41153781 PMC12561574

[82] Gu X Erb U Büchler MW Zöller M . Improved vaccine efficacy of tumor exosome compared to tumor lysate loaded dendritic cells in mice. Int J Cancer. (2015) 136:E74–84. doi: 10.1002/IJC.29100 25066479

[83] Dobrovolskienė N Pašukonienė V Darinskas A Kraśko JA Žilionytė K Mlynska A . Tumor lysate-loaded bacterial ghosts as a tool for optimized production of therapeutic dendritic cell-based cancer vaccines. Vaccine. (2018) 36:4171–80. doi: 10.1016/j.vaccine.2018.06.016 29895501

[84] Rainone V Martelli C Ottobrini L Biasin M Borelli M Lucignani G . Immunological characterization of whole tumour lysate-loaded dendritic cells for cancer immunotherapy. PLoS One. (2016) 11. doi: 10.1371/JOURNAL.PONE.0146622 26795765 PMC4721657

[85] Shi GN Zhang CN Xu R Niu JF Song HJ Zhang XY . Enhanced antitumor immunity by targeting dendritic cells with tumor cell lysate-loaded chitosan nanoparticles vaccine. Biomaterials. (2017) 113:191–202. doi: 10.1016/j.biomaterials.2016.10.047 27816821

[86] Gitlitz BJ Belldegrun AS Zisman A Chao DH Pantuck AJ Hinkel A . A pilot trial of tumor lysate-loaded dendritic cells for the treatment of metastatic renal cell carcinoma. J Immunother. (2003) 26:412–9. doi: 10.1097/00002371-200309000-00004 12973030

[87] Matsushita H Enomoto Y Kume H Nakagawa T Fukuhara H Suzuki M . A pilot study of autologous tumor lysate-loaded dendritic cell vaccination combined with sunitinib for metastatic renal cell carcinoma. J Immunother Cancer. (2014) 2. doi: 10.1186/S40425-014-0030-4 25694811 PMC4331924

[88] Salcedo M Bercovici N Taylor R Vereecken P Massicard S Duriau D . Vaccination of melanoma patients using dendritic cells loaded with an allogeneic tumor cell lysate. Cancer Immunol Immunother. (2006) 55:819–29. doi: 10.1007/S00262-005-0078-6 16187085 PMC11030805

[89] Zhu T Li Y Wang Y Li D . The application of dendritic cells vaccines in tumor therapy and their combination with biomimetic nanoparticles. Vaccines. (2025) 13. doi: 10.3390/VACCINES13040337 40333202 PMC12031636

[90] Zhang MH Scotland BL Jiao Y Slaby EM Truong N Cottingham AL . Lipid-polymer hybrid nanoparticles utilize B cells and dendritic cells to elicit distinct antigen-specific CD4+ and CD8+ T cell responses. ACS Appl Bio Mater. (2024) 7:4818–30. doi: 10.1021/ACSABM.3C00229 37219857 PMC10665545

[91] Sunoqrot S Abdel Gaber SA Abujaber R Al-Majawleh M Talhouni S . Lipid- and polymer-based nanocarrier platforms for cancer vaccine delivery. ACS Appl Bio Mater. (2024) 7:4998–5019. doi: 10.1021/ACSABM.3C00843 38236081

[92] Rosalia RA Cruz LJ van Duikeren S Tromp AT Silva AL Jiskoot W . CD40-targeted dendritic cell delivery of PLGA-nanoparticle vaccines induce potent anti-tumor responses. Biomaterials. (2015) 40:88–97. doi: 10.1016/J.BIOMATERIALS.2014.10.053 25465442

[93] Qin YT Liu XH An JX Liang JL Li CX Jin XK . Dendritic cell-based in situ nanovaccine for reprogramming lipid metabolism to boost tumor immunotherapy. ACS Nano. (2023) 17:24947–60. doi: 10.1021/ACSNANO.3C06784 38055727

[94] Yang W Zhu G Wang S Yu G Yang Z Lin L . In situ dendritic cell vaccine for effective cancer immunotherapy. ACS Nano. (2019) 13:3083–94. doi: 10.1021/ACSNANO.8B08346 30835435

[95] Hanlon DJ Aldo PB Devine L Alvero AB Engberg AK Edelson R . Enhanced stimulation of anti-ovarian cancer CD8(+) T cells by dendritic cells loaded with nanoparticle encapsulated tumor antigen. Am J Reprod Immunol. (2011) 65:597–609. doi: 10.1111/J.1600-0897.2010.00968.X 21241402 PMC3082607

[96] Prasad S Cody V Saucier-Sawyer JK Saltzman WM Sasaki CT Edelson RL . Polymer nanoparticles containing tumor lysates as antigen delivery vehicles for dendritic cell-based anti-tumor immunotherapy. Nanomedicine. (2010) 7:1. doi: 10.1016/J.NANO.2010.07.002 20692374 PMC3073408

[97] Srinivasan MK Prasad M . Recent advances in tumor targeted polymeric nanoparticles for HNC treatment: Enhancing therapeutic efficacy via engineered and biocompatible drug delivery systems. J Oral Biol Craniofacial Res. (2025) 15:1316. doi: 10.1016/J.JOBCR.2025.08.012 40895268 PMC12395162

[98] Zhang Z Tongchusak S Mizukami Y Kang YJ Ioji T Touma M . Induction of anti-tumor cytotoxic T cell responses through PLGA-nanoparticle mediated antigen delivery. Biomaterials. (2011) 32:3666–78. doi: 10.1016/j.biomaterials.2011.01.067 21345488

[99] Zhang C Shi G Zhang J Song H Niu J Shi S . Targeted antigen delivery to dendritic cell via functionalized alginate nanoparticles for cancer immunotherapy. J Control Release. (2017) 256:170–81. doi: 10.1016/j.jconrel.2017.04.020 28414151

[100] Xiang J Xu L Gong H Zhu W Wang C Xu J . Antigen-loaded upconversion nanoparticles for dendritic cell stimulation, tracking, and vaccination in dendritic cell-based immunotherapy. ACS Nano. (2015) 9:6401–11. doi: 10.1021/ACSNANO.5B02014 26028363

[101] Gilboa E Vieweg J . Cancer immunotherapy with mRNA-transfected dendritic cells. Immunol Rev. (2004) 199:251–63. doi: 10.1111/J.0105-2896.2004.00139.X 15233739

[102] Kyte JA Gaudernack G . Immuno-gene therapy of cancer with tumour-mRNA transfected dendritic cells. Cancer Immunol Immunother. (2006) 55:1432–42. doi: 10.1007/S00262-006-0161-7 16612595 PMC11030124

[103] Ni J Song J Wang B Hua H Zhu H Guo X . Dendritic cell vaccine for the effective immunotherapy of breast cancer. BioMed Pharmacother. (2020) 126. doi: 10.1016/j.biopha.2020.110046 32145586

[104] Plebanek MP Sturdivant M Devito NC Hanks BA . Role of dendritic cell metabolic reprogramming in tumor immune evasion. Int Immunol. (2020) 32:485–91. doi: 10.1093/INTIMM/DXAA036 32449776 PMC7318778

[105] Li H Wang C Yu J Cao S Wei F Zhang W . Dendritic cell-activated cytokine-induced killer cells enhance the anti-tumor effect of chemotherapy on non-small cell lung cancer in patients after surgery. Cytotherapy. (2009) 11:1076–83. doi: 10.3109/14653240903121252 19929470

[106] Van De Laar L Coffer PJ Woltman AM . Regulation of dendritic cell development by GM-CSF: molecular control and implications for immune homeostasis and therapy. Blood. (2012) 119:3383–93. doi: 10.1182/BLOOD-2011-11-370130 22323450

[107] Malik JA Khan M Hani U Rani VI Bhosale RR . Nanotechnology-based cytokine delivery strategies in gastrointestinal cancers. Cytokine Growth Factor Rev. (2025) 86:222–37. doi: 10.1016/j.cytogfr.2025.11.002 41242181

[108] Clayton G Toffoli EC de Gruijl TD van Kooij Y . Dendritic cell immunotherapy advances for solid tumors: vaccination and modulation. Cell Rep Med. (2025) 6(11):102412. doi: 10.1016/j.xcrm.2025.102412 41086813 PMC12711664

[109] Butterfield LH . Dendritic cells in cancer immunotherapy clinical trials: are we making progress? Front Immunol. (2013) 4. doi: 10.3389/FIMMU.2013.00454 24379816 PMC3861778

[110] Anguille S Smits EL Lion E Van Tendeloo VF Berneman ZN . Clinical use of dendritic cells for cancer therapy. Lancet Oncol. (2014) 15. doi: 10.1016/S1470-2045(13)70585-0 24872109

[111] Lodge PA Jones LA Bader RA Murphy GP Salgaller ML . Dendritic cell-based immunotherapy of prostate cancer: immune monitoring of a phase II clinical trial. Cancer Res. (2000) 60:829–33. doi: 10.1615/critrevimmunol.v18.i1-2.120 10706088

[112] Jiang W Wang Z Luo Q Dai Z Zhu J Tao X . Combined immunotherapy with dendritic cells and cytokine-induced killer cells for solid tumors: a systematic review and meta-analysis of randomized controlled trials. J Transl Med. (2024) 22. doi: 10.1186/S12967-024-05940-Y 39707416 PMC11662567

[113] Bapsy PP Sharan B Kumar C Das RP Rangarajan B Jain M . Open-label, multi-center, non-randomized, single-arm study to evaluate the safety and efficacy of dendritic cell immunotherapy in patients with refractory solid Malignancies, on supportive care. Cytotherapy. (2014) 16:234–44. doi: 10.1016/j.jcyt.2013.11.013 24438902

[114] Yamanaka R Homma J Yajima N Tsuchiya N Sano M Kobayashi T . Clinical evaluation of dendritic cell vaccination for patients with recurrent glioma: results of a clinical phase I/II trial. Clin Cancer Res. (2005) 11:4160–7. doi: 10.1158/1078-0432.CCR-05-0120 15930352

[115] Dillman RO Cornforth AN Depriest C McClay EF Amatruda TT De Leon C . Tumor stem cell antigens as consolidative active specific immunotherapy: a randomized phase II trial of dendritic cells versus tumor cells in patients with metastatic melanoma. J Immunother. (2012) 35:641–9. doi: 10.1097/CJI.0B013E31826F79C8 22996370

[116] Miwa S Nishida H Tanzawa Y Takeuchi A Hayashi K Yamamoto N . Phase 1/2 study of immunotherapy with dendritic cells pulsed with autologous tumor lysate in patients with refractory bone and soft tissue sarcoma. Cancer. (2017) 123:1576–84. doi: 10.1002/CNCR.30606 28241093

[117] Chang CN Huang YC Yang DM Kikuta K Wei KJ Kubota T . A phase I/II clinical trial investigating the adverse and therapeutic effects of a postoperative autologous dendritic cell tumor vaccine in patients with Malignant glioma. J Clin Neurosci. (2011) 18:1048–54. doi: 10.1016/j.jocn.2010.11.034 21715171

[118] Rodriguez J Castañón E Perez-Gracia JL Rodriguez I Viudez A Alfaro C . A randomized phase II clinical trial of dendritic cell vaccination following complete resection of colon cancer liver metastasis. J Immunother Cancer. (2018) 6. doi: 10.1186/S40425-018-0405-Z 30268156 PMC6164167

[119] Itoh T Ueda Y Kawashima I Nukaya I Fujiwara H Fuji N . Immunotherapy of solid cancer using dendritic cells pulsed with the HLA-A24-restricted peptide of carcinoembryonic antigen. Cancer Immunol Immunother. (2002) 51:99–106. doi: 10.1007/S00262-001-0257-Z 11904734 PMC11032765

[120] Lee WC Wang HC Hung CF Huang PF Lia CR Chen MF . Vaccination of advanced hepatocellular carcinoma patients with tumor lysate-pulsed dendritic cells: a clinical trial. J Immunother. (2005) 28:496–504. doi: 10.1097/01.CJI.0000171291.72039.E2 16113606

[121] Wang S Song Y Shi Q Qiao G Zhao Y Zhou L . Safety of dendritic cell and cytokine-induced killer (DC-CIK) cell-based immunotherapy in patients with solid tumor: a retrospective study in China. Am J Cancer Res. (2023) 13:4767. 37970341 PMC10636667

[122] Gerhard GM Bill R Messemaker M Klein AM Pittet MJ . Tumor-infiltrating dendritic cell states are conserved across solid human cancers. J Exp Med. (2021) 218. doi: 10.1084/JEM.20200264 33601412 PMC7754678

[123] Maeng HM Moore BN Bagheri H Steinberg SM Inglefield J Dunham K . Phase I clinical trial of an autologous dendritic cell vaccine against HER2 shows safety and preliminary clinical efficacy. Front Oncol. (2021) 11. doi: 10.3389/FONC.2021.789078 34976830 PMC8716407

[124] Han HS Aldrich AL Garg SK Weinfurtner RJ Nguyen JV Mo Q . Alteration of the tumor microenvironment with intratumoral dendritic cells before chemotherapy in ERBB2 breast cancer: a nonrandomized clinical trial. JAMA Oncol. (2025) 11:119–27. doi: 10.1001/JAMAONCOL.2024.5371 39636623 PMC11622104

[125] Sadeghzadeh M Bornehdeli S Mohahammadrezakhani H Abolghasemi M Poursaei E Asadi M . Dendritic cell therapy in cancer treatment; the state-of-the-art. Life Sci. (2020) 254. doi: 10.1016/j.lfs.2020.117580 32205087

[126] van Gulijk M Dammeijer F Aerts JGJV Vroman H . Combination strategies to optimize efficacy of dendritic cell-based immunotherapy. Front Immunol. (2018) 9. doi: 10.3389/FIMMU.2018.02759 30568653 PMC6289976

[127] Zanotta S Galati D De Filippi R Pinto A . Enhancing dendritic cell cancer vaccination: the synergy of immune checkpoint inhibitors in combined therapies. Int J Mol Sci. (2024) 25. doi: 10.3390/IJMS25147509 39062753 PMC11277144

[128] Garcin G De Koker S Van Parys A Wuest T Gerlo S Van der Heyden J . Delivering Type I interferon to dendritic cells empowers tumor eradication and immune combination treatments. Cancer Res. (2018) 78(2):463–474. doi: 10.1158/0008-5472.CAN-17-1980 29187401

[129] Cauwels A Van Lint S Paul F Garcin G De Koker S Van Parys A . Delivering type I interferon to dendritic cells empowers tumor eradication and immune combination treatments. Cancer Res. (2018) 78:463–74. doi: 10.1158/0008-5472.CAN-17-1980 29187401

[130] Baek S Kim CS Kim SB Kim YM Kwon SW Kim YM . Combination therapy of renal cell carcinoma or breast cancer patients with dendritic cell vaccine and IL-2: results from a phase I/II trial. J Transl Med. (2011) 9. doi: 10.1186/1479-5876-9-178 22013914 PMC3213212

[131] Bin Y Kusmartsev S Fengdong C Paolini M Nefedova Y Sotomayor E . Effective combination of chemotherapy and dendritic cell administration for the treatment of advanced-stage experimental breast cancer. Clin Cancer Res. (2003) 9:285–94. 12538481

[132] Garofano F Gonzalez-Carmona MA Skowasch D Schmidt-Wolf R Abramian A Hauser S . Clinical trials with combination of cytokine-induced killer cells and dendritic cells for cancer therapy. Int J Mol Sci. (2019) 20. doi: 10.3390/IJMS20174307 31484350 PMC6747410

[133] Zhong R Teng J Han B Zhong H . Dendritic cells combining with cytokine-induced killer cells synergize chemotherapy in patients with late-stage non-small cell lung cancer. Cancer Immunol Immunother. (2011) 60:1497–502. doi: 10.1007/S00262-011-1060-0 21681372 PMC11029021

[134] Butterfield LH Najjar YG . Immunotherapy combination approaches: mechanisms, biomarkers and clinical observations. Nat Rev Immunol. (2023) 24:399. doi: 10.1038/S41577-023-00973-8 38057451 PMC11460566

[135] Tesone AJ Svoronos N Allegrezza MJ Conejo-Garcia JR . Pathological mobilization and activities of dendritic cells in tumor-bearing hosts: challenges and opportunities for immunotherapy of cancer. Front Immunol. (2013) 4. doi: 10.3389/FIMMU.2013.00435 24339824 PMC3857526

[136] Cintolo JA Datta J Mathew SJ Czerniecki BJ . Dendritic cell-based vaccines: barriers and opportunities. Future Oncol. (2012) 8:1273. doi: 10.2217/FON.12.125 23130928 PMC4260651

[137] Fu C Ma T Zhou L Mi QS Jiang A . Dendritic cell-based vaccines against cancer: challenges, advances and future opportunities. Immunol Invest. (2022) 51:2133–58. doi: 10.1080/08820139.2022.2109486 35946383

[138] Mantia-Smaldone GM Chu CS . A review of dendritic cell therapy for cancer: progress and challenges. BioDrugs. (2013) 27:453–68. doi: 10.1007/S40259-013-0030-9 23592406

[139] Hájek R Butch AW . Dendritic cell biology and the application of dendritic cells to immunotherapy of multiple myeloma. Med Oncol. (2000) 17:2–15. doi: 10.1007/BF02826210 10713654

[140] Verheye E Melgar JB Deschoemaeker S Raes G Maes A De Bruyne E . Dendritic cell-based immunotherapy in multiple myeloma: challenges, opportunities, and future directions. Int J Mol Sci. (2022) 23. doi: 10.3390/IJMS23020904 35055096 PMC8778019

[141] Johnson P Rosendahl N Radford KJ . Conventional type 1 dendritic cells (cDC1) as cancer therapeutics: challenges and opportunities. Expert Opin Biol Ther. (2022) 22:465–72. doi: 10.1080/14712598.2022.1994943 34654337

[142] Hong S Li H Qian J Yang J Lu Y Yi Q . Optimizing dendritic cell vaccine for immunotherapy in multiple myeloma: tumour lysates are more potent tumour antigens than idiotype protein to promote anti-tumour immunity. Clin Exp Immunol. (2012) 170:167–77. doi: 10.1111/J.1365-2249.2012.04642.X 23039887 PMC3482363

[143] Bol KF Schreibelt G Gerritsen WR De Vries IJM Figdor CG . Dendritic cell-based immunotherapy: state of the art and beyond. Clin Cancer Res. (2016) 22:1897–906. doi: 10.1158/1078-0432.CCR-15-1399 27084743

